# Partitioning the Two-Leg Spin Ladder in Ba_2_Cu_1 – *x*_Zn_*x*_TeO_6_: From Magnetic Order through Spin-Freezing to Paramagnetism

**DOI:** 10.1021/acs.chemmater.2c02939

**Published:** 2023-03-22

**Authors:** Charlotte Pughe, Otto H. J. Mustonen, Alexandra S. Gibbs, Stephen Lee, Rhea Stewart, Ben Gade, Chennan Wang, Hubertus Luetkens, Anna Foster, Fiona C. Coomer, Hidenori Takagi, Edmund J. Cussen

**Affiliations:** † Department of Material Science and Engineering, University of Sheffield, Sheffield S1 3JD, United Kingdom; ‡ School of Chemistry, University of Birmingham, Edgbaston, Birmingham B15 2TT, United Kingdom; § School of Chemistry, University of St Andrews, St Andrews KY16 9ST , United Kingdom; ∥ ISIS Pulsed Neutron and Muon Source, STFC Rutherford Appleton Laboratory, Didcot OX11 0QX, United Kingdom; ⊥ Max Planck Institute for Solid State Research, Heisenbergstrasse 1, 70569 Stuttgart, Germany; # School of Physics and Astronomy, St Andrews KY16 9SS, United Kingdom; ∇ Paul Scherrer Institute, Forschungsstrasse 111, 5232 Villigen PSI, Switzerland; ○ Department of Chemistry, University of Sheffield, Sheffield S3 7HF, United Kingdom; ◆ Echion Technologies, Sawston, Cambridge CB22 3FG, United Kingdom; ¶ Department of Physics, University of Tokyo, Tokyo 113-0013, Japan; & Institute for Functional Matter and Quantum Technologies, University of Stuttgart, 70569 Stuttgart, Germany

## Abstract

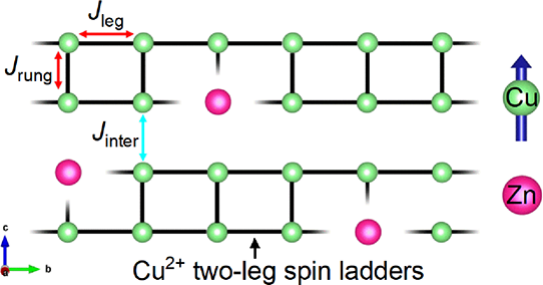

Ba_2_CuTeO_6_ has attracted significant attention as it contains a two-leg spin ladder of Cu^2+^ cations that lies in close proximity to a quantum critical point. Recently, Ba_2_CuTeO_6_ has been shown to accommodate chemical substitutions, which can significantly tune its magnetic behavior. Here, we investigate the effects of substitution for non-magnetic Zn^2+^ impurities at the Cu^2+^ site, partitioning the spin ladders. Results from bulk thermodynamic and local muon magnetic characterization on the Ba_2_Cu_1 – *x*_Zn_*x*_TeO_6_ solid solution (0 ≤ *x* ≤ 0.6) indicate that Zn^2+^ partitions the Cu^2+^ spin ladders into clusters and can be considered using the percolation theory. As the average cluster size decreases with increasing Zn^2+^ substitution, there is an evolving transition from long-range order to spin-freezing as the critical cluster size is reached between *x* = 0.1 to *x* = 0.2, beyond which the behavior became paramagnetic. This demonstrates well-controlled tuning of the magnetic disorder, which is highly topical across a range of low-dimensional Cu^2+^-based materials. However, in many of these cases, the chemical disorder is also relatively strong in contrast to Ba_2_CuTeO_6_ and its derivatives. Therefore, Ba_2_Cu_1 – *x*_Zn_*x*_TeO_6_ provides an ideal model system for isolating the effect of defects and segmentation in low-dimensional quantum magnets.

## 1 Introduction

Copper oxides are excellent hosts for unusual magnetic phenomena. This is due to the quantum spin *S* = 1/2 of Cu^2+^ cations combined with the strong Jahn–Teller effect, which leads to co-operative orbital ordering that effectively lowers the dimensionality of the interactions between the Cu^2+^ spins. The quantum spin and low dimensionality enhances quantum effects and can give rise to a range of exotic quantum magnetic phases and transitions, many of which are of technological value.^1^ As a result, copper-based transition metal oxides such as perovskites are desirable models to study existing and discover new low-dimensional quantum phenomena, e.g., high temperature superconductivity, frustrated magnetism, and quantum magnetic transitions.^2−8^

**Figure 1:**
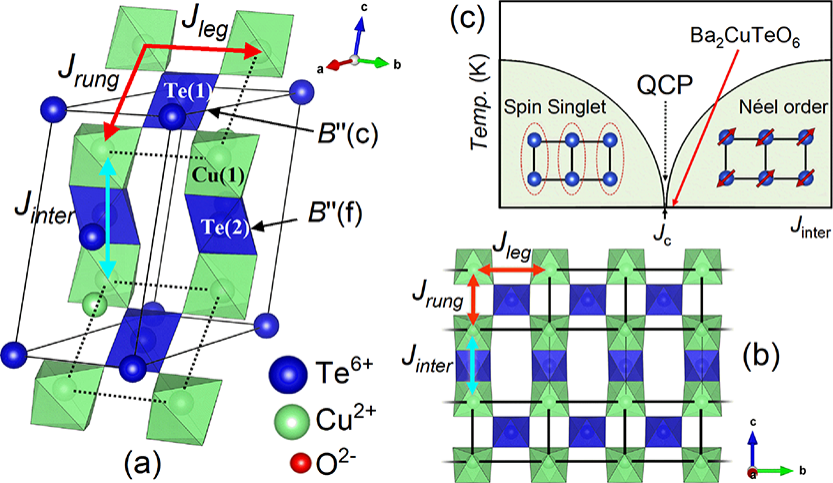
(a) Monoclinic structure of Ba_2_CuTeO_6_ showing the 12R hexagonal stacking sequence. The intra-ladder (*J*_leg_ and *J*_rung_) interactions between the Cu^2+^ cations (colored green) are indicated by the red arrows. The inter-ladder interaction *J*_inter_ through the face-sharing CuO_6_-TeO_6_-CuO_6_ trimer is indicated by the blue arrow. (b) Two-leg spin ladder structure of Cu^2+^ cations in Ba_2_CuTeO_6_ viewed along the *a* axis. (c) Two-leg spin ladder phase diagram. The red arrow shows that Ba_2_CuTeO_6_ lies close to the quantum critical point (QCP) on the Nèel ordered side of the phase diagram.

The two-leg spin ladder in Ba_2_CuTeO_6_ is an example of a low-dimensional copper perovskite. The 12R hexagonal perovskite structure of Ba_2_CuTeO_6_ has face-sharing CuO_6_-TeO_6_-CuO_6_ trimers linked by corner-sharing TeO_6_ units (Figure 1a).^9^ Through Cu-O-Te-O-Cu superexchange, this creates two-leg Cu^2+^ spin ladders along the *b* axis of the monoclinic crystal structure, wherein the intra-ladder superexchange interactions are the *J*_leg_ and *J*_rung_ interactions shown by the red arrows in Figure 1b. A weak inter-ladder exchange (*J*_inter_) occurs through the face-sharing trimers, creating a highly quasi-two-dimensional system.^10^ This system has attracted interest as it lies very close to the quantum critical point (QCP) on the Nèel ordered side of the two-leg spin ladder phase diagram shown in Figure 1c.^11−13^ QCPs are electronic phase transitions at absolute zero, and they occur in a range of technologically important materials (e.g., superconductors, insulators, and semiconductors).^14−17^

We have recently demonstrated that the intra-ladder interactions in Ba_2_CuTeO_6_ can be site-selectively tuned through W^6+^ substitution.^18^ W^6+^ is almost exclusively substituted for Te^6+^ at the corner-sharing site (*B*″(c)) rather than the face-sharing trimer site (*B*″(f)) indicated in Figure 1. Through the *d*^10^/*d*^0^ effect, the competing *d*^10^ Te^6+^ and *d*^0^ W^6+^ interactions strongly suppress the *J*_rung_, while the *J*_leg_ is slightly strengthened.^18,19^ This tunes the system from a spin ladder toward a spin chain, further reducing the dimensionality of the Cu^2+^ interactions. It is possible that the dimensionality of the two-leg spin ladder could be modified from another perspective. Instead of between ladders, substitution could be performed directly within the ladder at the Cu^2+^ site. Non-magnetic impurities, whether intrinsic or purposefully introduced, are an important consideration when synthesizing magnetic materials and have been considered using percolation theory in square lattices, spin chains, and spin ladders.^20,21^ In a two-leg spin ladder, any finite impurities will segment the ladder into clusters. This is due to the fact that a ladder is a one-dimensional system, and two neighboring non-magnetic impurities linked by a “rung” will create a break in the ladder interactions. The size of the clusters is controlled by the level of non-magnetic impurities.^21^

Non-magnetic Zn^2+^ impurities have been studied in two-leg spin ladders previously. Examples include Sr(Cu_1 – *x*_Zn_*x*_)_2_O_3_, Bi(Cu_1 – *x*_Zn_*x*_)_2_PO_6_, and (C_7_H_10_N)_2_Cu_1 – *x*_Zn_*x*_(Br)_4_.^22−29^ These two-leg spin ladders lie on the spin singlet side of the two-leg spin ladder phase diagram, where *J*_inter_ is weak, creating near-isolated spin ladders. As expected, the introduction of Zn^2+^ creates “free” Cu^2+^ spins as singlet dimers are broken by the removal of Cu^2+^.^23^ Unexpectedly, antiferromagnetic ordering has also been observed for Sr(Cu_1 – *x*_Zn_*x*_)_2_O_3_ and Bi(Cu_1 – *x*_Zn_*x*_)_2_PO_6_ with low Zn^2+^ concentrations (*x* = 0.01–0.02).^24,25,29^ It is proposed that antiferromagnetic order arises from Cu^2+^ moments generated in the vicinity of the Zn^2+^ impurity.^28^ The Cu^2+^ moments are independent of geometry and create antiferromagnetic correlations.^29^ Theoretical calculations suggest that the extended Cu^2+^ spin ladder interactions are not destroyed and only the local Cu^2+^ singlets are affected.^30^ The effect of Zn^2+^ impurities in Nèel ordered two-leg spin ladders with stronger *J*_inter_ interactions remains experimentally unexplored. To investigate, a solid solution of Ba_2_Cu_1 – *x*_Zn_*x*_TeO_6_ (0 ≤ *x* ≤ 0.6) was prepared and analyzed using a range of structural and magnetic characterization techniques.

## 2 Experimental Section

### 2.1 Synthesis

Polycrystalline powders of Ba_2_Cu_1 – *x*_Zn_*x*_TeO_6_, 0 ≤ *x* ≤ 0.6, were prepared by mixing high-purity BaCO_3_ (99.997%), CuO (99.9995%), ZnO (99.99%), and TeO_2_ (99.995%). The reactant mixture was pressed into a pellet and calcined in air for 12 h at 900 °C. Calcined pellets were re-ground and pressed before heating at 1050–1100 °C under a flow of oxygen for 24 h. A total of 72 h (3 × 24 h) was required to achieve phase purity in all samples.

### 2.2 X-ray and Neutron Diffraction

A Rigaku Miniflex diffractometer (Cu K_α1_/ K_α2_ (λ = 1.5405 and 1.5443 Å)) monitored the sample purity during the reaction. Neutron diffraction data were collected on the time-of-flight diffractometer HRPD at the ISIS Neutron and Muon Source.^31,32^ The data were collected at ambient temperature in a standard time-of-flight window of 30–130 ms with the sample contained in standard cylindrical vanadium cans. Data were analyzed using Rietveld refinement as implemented in GSAS, TOPAS Academic v7, and PIEFACE for polyhedral distortions.^33−35^

### 2.3 Inductively Coupled Plasma–Optical Emission Spectroscopy

ICP-OES was performed on *x* = 0.1–0.6 samples to determine the relative percentage of Cu^2+^ and Zn^2+^ in the samples. Powder samples were digested in an aqua regia mixture at 150 °C before being analyzed by a Spectrogreen FMX46 ICP-OES where, upon ionization, the percentage Cu^2+^ and Zn^2+^ in each sample was determined from the light emitted at wavelengths of 324.754 nm (Cu) and 213.856 nm (Zn) using an optical spectrometer.

### 2.4 Magnetic Susceptibility

Measurements were performed using a Quantum Design MPMS3 SQUID magnetometer. The DC susceptibility (χ vs *T*) was measured between 2 and 300 K in both zero-field cooled (ZFC) and field-cooled (FC) modes using a 1000 Oe external field. AC susceptibility (χ_AC_^′^ vs *T*) measurements were performed on the *x* = 0.1, 0.2, and 0.3 samples. Using a weak DC field of 25 Oe and an AC field of 5 Oe, the AC susceptibility was measured from 2 to 100 K in a frequency range of 10 to 467 Hz.

### 2.5 Heat Capacity

A Quantum design PPMS was used to perform heat capacity measurements. Shards of sintered pellets weighing ∼10 mg were placed onto the sample puck using Apiezon N grease, and the heat capacity was measured using the thermal relaxation method between 2 and 100 K in the zero field. The contribution of the grease and puck was subtracted from the total measurement to give the heat capacity of the sample.

### 2.6 Muon Spin Relaxation

Muon experiments were performed at the Paul Scherrer Institut (PSI) using the GPS beamline. Approximately 1 g of polycrystalline powder (*x* = 0, 0.1, 0.2, and 0.3) was loaded into a silver foil packet and secured onto the sample fork. The sample fork was inserted into the muon beam and cooled to 1.5 K using a cryostat. Zero-field (ZF), transverse-field (TF), and longitudinal-field (LF) muon spin relaxation measurements were performed between 1.5 and 20 K. The data were analyzed using musrfit.^36^

## 3 Results

### 3.1 Crystal Structure

**Figure 2:**
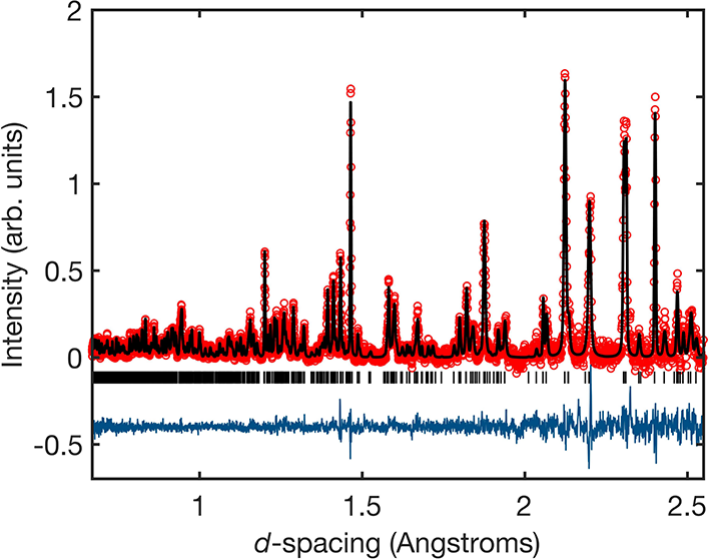
Rietveld refinement of the monoclinic *x* = 0.1 model using the 300 K HRPD neutron diffraction data for Ba_2_Cu_0.9_Zn_0.1_TeO_6_ (*R*_wp_(%) = 6.41 and χ^2^ = 2.924).

Our high resolution neutron diffraction data confirm that the same monoclinic *C*2/*m* crystal structure is present across the series 0 ≤*x* ≤ 0.6 at *T* = 300 K. Figure 2 shows an example of the Rietveld refinement for *x* = 0.1, wherein all the Bragg peaks are described well by the refined *C*2/*m* model shown in Table S1. It should be noted that the parent *x* = 0 compound has a weak transition to a triclinic *P*1̅ phase at *T* = 287 K, and the *x* = 1 composition is rhombohedral (*R*3̅*m*).^9,10^*C*2/*m* to *P*1̅ distortions could be observed for Ba_2_Cu_1 – *x*_Zn_*x*_TeO_6_ upon cooling. This distortion will have a minor effect on the structure and interactions as the *C*2/*m* and *P*1̅ models are very similar. Consequently, the high-temperature *C*2/*m* structure can be used to model the magnetic interactions in the low-temperature *P*1̅ structure of Ba_2_CuTeO_6_.^12^

Substitution of Zn^2+^ for Cu^2+^ leads to a systematic reduction in the *a* lattice parameter, an increase in *b* and *c*, and an increase in the monoclinic β angle. This results from a weakening of the strength of the cooperative Jahn–Teller distortion with increasing *x*. Due to the significant irregularity in the shape and bond lengths of the (Cu,Zn)O_6_ octahedra, this may be better quantified through minimum bounding ellipsoid analysis^34^ than by the investigation of specific bond lengths and angles. The main parameter of interest from this calculation is the magnitude of the largest ellipsoidal principal axis (effectively the Jahn–Teller axis), *R*_1_. As *x* varies from 0 to 0.2 to 0.4, for example, the magnitude of this parameter decreases from 2.371 Å to 2.356 and 2.343 Å. In addition, the variance of the principal axes indicates the overall strength of the distortion from an ideal polyhedron. As expected, this reduces monotonically with increasing *x* with σ(*R*)_*x* = 0_ = 0.192, σ(*R*)_*x* = 0.2_ = 0.179, and σ(*R*)_*x* = 0.4_ = 0.167.

Given the similar Cu^2+^ and Zn^2+^ X-ray and neutron scattering lengths, ICP-OES was used to confirm the samples’ stoichiometries. The ICP-OES results gave the percentages of Cu^2+^ and Zn^2+^ in each composition. The percentage of Zn^2+^ was divided by the total percentages of Cu^2+^ and Zn^2+^ in each sample. This gave the proportion of Zn^2+^ in the sample as a decimal where the total amount of Cu^2+^ + Zn^2+^ = 1, and the Cu^2+^ portion was found by Cu^2+^ = 1- Zn^2+^. Table 1 shows that the Zn^2+^ portion incrementally increases by ∼0.1 for each *x* = 0.1 increase in the Zn^2+^ concentration, while the Cu^2+^ portion decreases by ∼0.1. This agrees with the sample stoichiometry of the *x* = 0.1–0.6 samples, confirming no elemental losses.

**Table 1:** Results from ICP-OES Measurements of the Ba_2_Cu_*x*_Zn_1 – *x*_TeO_6_*x* = 0.1–0.6 Samples Showing the Amount of Zn^2+^ and Cu^2+^ in Each Sample as a Proportion of the Total Amount of Cu^2+^ and Zn^2+^, Where Cu^2+^ + Zn^2+^ = 1

*x*	Zn^2+^	Cu^2+^
0.1	0.1068(3)	0.893(3)
0.2	0.2116(6)	0.788(2)
0.3	0.312(3)	0.688(6)
0.4	0.417(4)	0.583(6)
0.5	0.516(3)	0.484(2)
0.6	0.621(2)	0.388(1)

### 3.2 DC Susceptibility

**Figure 3:**
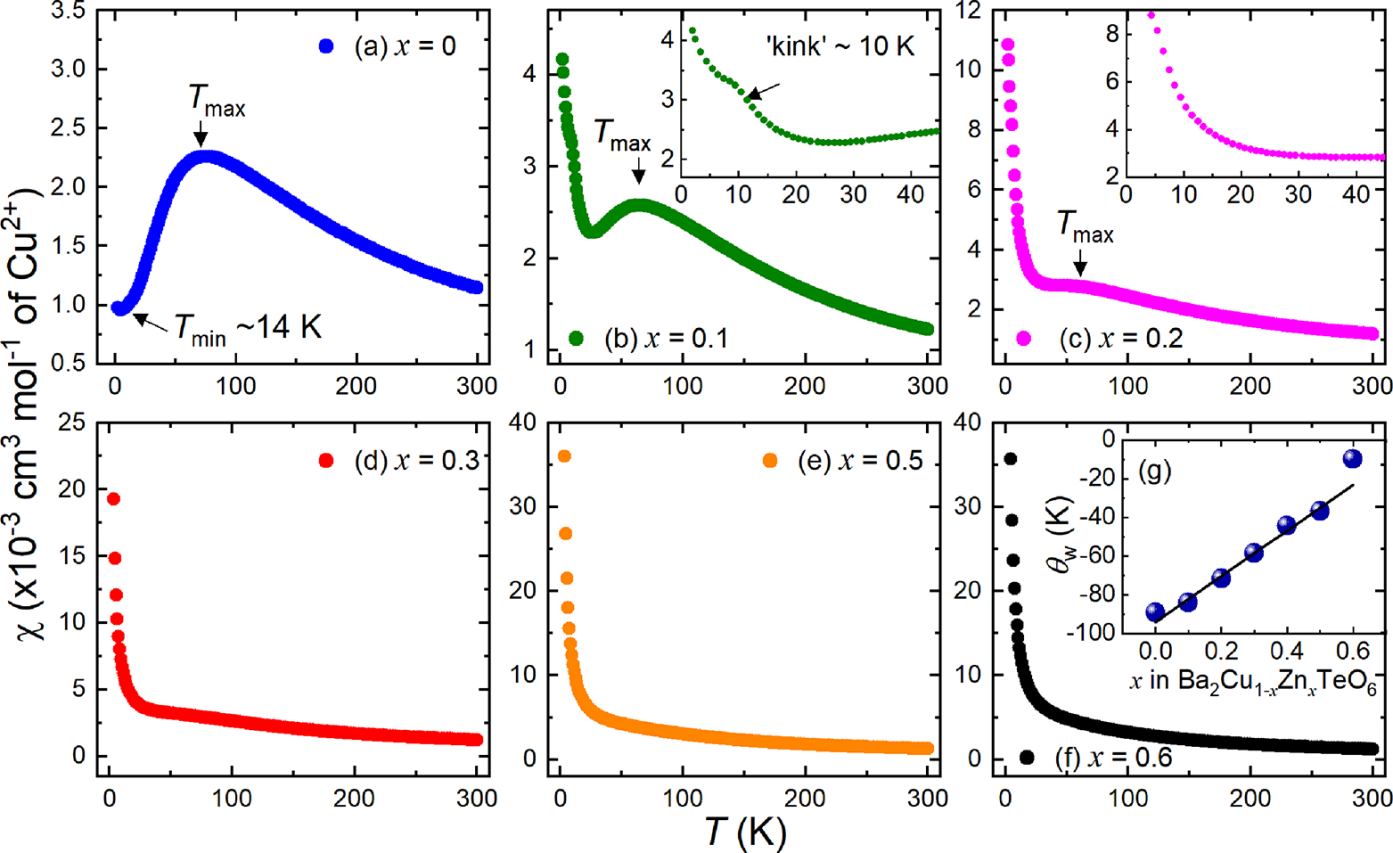
DC susceptibility data of the (a) *x* = 0, (b) *x* = 0.1, (c) *x* = 0.2, (d) *x* = 0.3, (e) *x* = 0.5, and (f) *x* = 0.6 Ba_2_Cu_*x*_Zn_1 – *x*_TeO_6_ samples. χ is scaled to cm^3^ mol^–1^ of Cu^2+^ to reflect dilution of the Cu^2+^ site. The χ vs *T* data of *x* = 0.4 was identical to other samples beyond *x* ≥ 0.3. The position of *T*_max_ and/or *T*_min_ are indicated in the χ vs *T* curves where appropriate. Expansions of the low-temperature data are shown in panels (b) and (c). There is a clear “kink” at ∼10 K in the *x* = 0.1 curve that is not visible in the *x* ≥ 0.2 curves. (g) Weiss constant (θ_W_) plotted as a function of *x* in Ba_2_Cu_1 – *x*_Zn_*x*_TeO_6_. θ_W_ increases linearly as *x* increases, showing large weakening of the magnetic interactions.

Figure 3 shows the χ vs *T* data for *x* = 0, 0.1, 0.2, 0.3, 0.5 and 0.6. No ZFC and FC divergence was observed for any of the samples. There are clear changes in the features of the χ vs *T* curve as Zn^2+^ is introduced to Ba_2_CuTeO_6_. To most clearly show the effect of dilution of Cu^2+^ by Zn^2+^, the susceptibility has been scaled to cm^3^ mol^–1^ of Cu^2+^. Panel (a) shows the χ vs *T* curve of *x* = 0 has a broad maximum of about *T*_max_ ≈ 74 K, below which the susceptibility decreases leading to a low-temperature upturn of about *T*_min_ ≈ 14 K. *T*_max_ represents the establishment of short-range ladder interactions. The low temperature upturn is thought to indicate entry to the Nèel ordered state but is not a classical indication of antiferromagnetic order.^10,18^ Therefore, the upturn cannot be assumed to be the position of *T*_N_. Instead, magnetic ordering has been confirmed using other methods and places *T*_N_ at 14.1 K for Ba_2_CuTeO_6_.^5^

The introduction of 10% Zn^2+^ (Figure 3b) causes a sharp rise in the low-temperature upturn feature and a shift in *T*_max_ toward lower temperatures. The decrease in *T*_max_ (Table 2) indicates weakening of the short-range interactions. The expansion in panel (b) shows a visible “kink” in the low-temperature data at 10 K, close to the position of the *T*_min_ upturn in *x* = 0. Beyond *x* = 0.1, there is no visible “kink” in the low-temperature data (see expansion in Figure 3c). The low-temperature susceptibility continues to grow, and the *T*_max_ feature transitions into a large paramagnetic tail. The inverse 1/χ vs *T* data between 150 and 300 K were fitted using the Curie–Weiss law (see Supplementary Figure S15). Table 2 shows the values of the Curie constant (*C*), Weiss constant (θ_W_), and effective magnetic moment (μ_eff_). The linear change in θ_W_ (plotted in Figure 3g) from −89.3(4) K for *x* = 0 to a value of −9.9(5) K for *x* = 0.6 shows a large weakening of the antiferromagnetic interactions. Table 2 shows that the μ_eff_ is close to the previously reported value for Ba_2_CuTeO_6_ and Ba_2_CuTe_1 – *x*_W_*x*_O_6_.^10,18^

**Table 2:** Results from DC χ vs *T* Data for Ba_2_Cu_*x*_Zn_1 – *x*_TeO_6_ (0 ≤ *x* ≤ 0.6)

*x*	*T*_max_ (K)	*C* (cm^3^ K mol^–1^)	θ_W_ (K)	μ_eff_ (μ_B_ per Cu^2+^)
0	73.7	0.4450(7)	–89.3(4)	1.890(5)
0.1	64	0.4688(8)	–84.0(3)	1.936(2)
0.2	∼57	0.4438(4)	–71.70(9)	1.8840(8)
0.3		0.4365(7)	–58.6(2)	1.869(1)
0.4		0.4291(7)	–44.3(2)	1.852(1)
0.5		0.4214(6)	–36.6(2)	1.835(1)
0.6		0.3751(8)	–9.9(3)	1.732(2)

**Figure 4:**
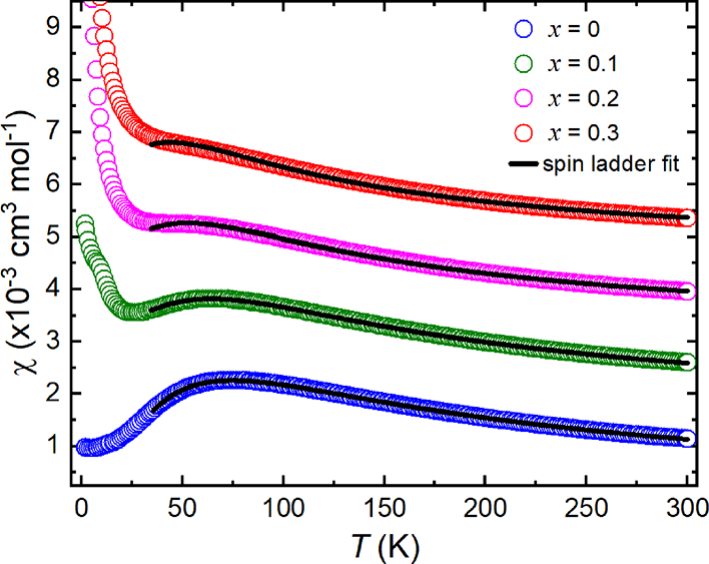
Modeling the χ vs *T* data of *x* = 0, 0.1, and 0.2 using the QMC isolated two-leg spin ladder model. The fits are shown by the solid black lines. The data are offset along the *y* axis. The isolated two-leg spin ladder model provides a good description of the *x* = 0 and *x* = 0.1 data, allowing extraction of the *J*_leg_, *J*_rung_/*J*_leg_, and *g* fitting parameters. The fit to the *x* = 0.2 and *x* = 0.3 data shows that the model increasingly fails to described the suppressing *T*_max_ feature.

The χ vs *T* data for 0 ≤ *x* ≤ 0.3 were modeled using the isolated two-leg spin ladder model between 35 and 300 K. The model is based on Quantum Monte Carlo (QMC) simulations of isolated two-leg spin ladders and has been employed to model Ba_2_CuTeO_6_ and Ba_2_CuTe_1 – *x*_W_*x*_O_6_ previously.^10,18,22^ The fitting parameters, *J*_leg_, *J*_rung_/*J*_leg_, and Landè *g*-factor, for *x* = 0 were near identical to previous reports: *J*_leg_ = 89.2(3) K, *J*_rung_/*J*_leg_ = 0.972(6), and *g* = 2.231(2).^10,18^ The *x* = 0.1 data could be described using the spin ladder model but, as shown in Figure 4, began to fail for *x* = 0.2 as the *T*_max_ feature is suppressed. The model completely fails for *x* = 0.3, indicating a change from spin ladder behavior. This can be seen by comparing the fits shown by the solid black lines in Figure 4. Hence, accurate fitting parameters could only be obtained for *x* = 0.1 and suggest slight strengthening of the *J*_leg_ = 99.5(2) K interaction compared to *x* = 0. The *J*_rung_/*J*_leg_ = 0.17(2) ratio is significantly reduced from near unity in the *x* = 0 compound, showing strong suppression of the *J*_rung_ interaction. This agrees with the values of θ_W_ and μ_eff_, which suggest weakening of the overall intra-ladder interactions in *x* = 0.1.

### 3.3 AC Susceptibility

**Figure 5:**
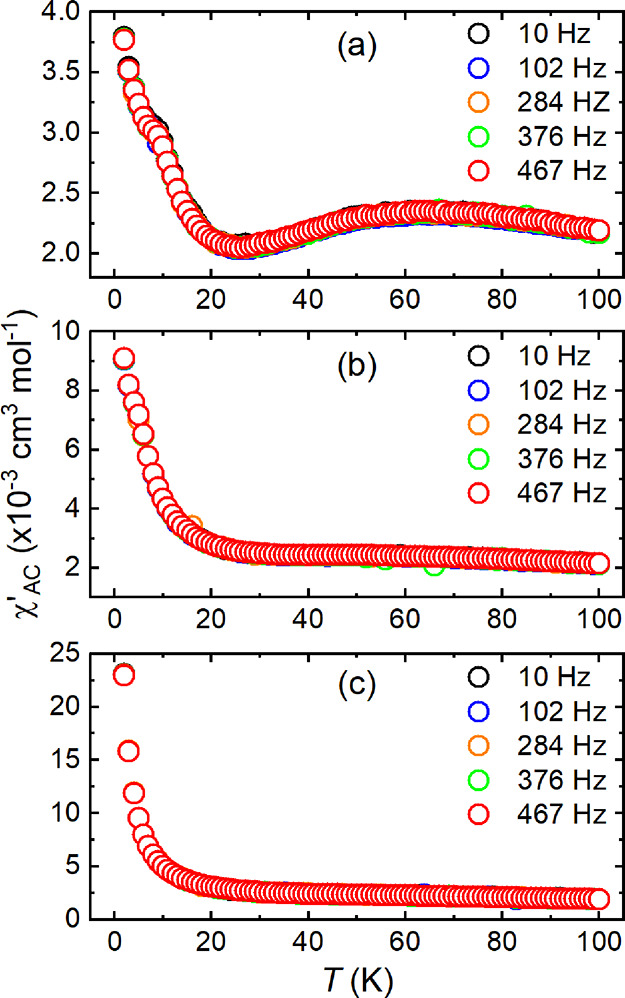
AC susceptibility data of the (a) *x* = 0.1, (b) *x* = 0.2, and (c) *x* = 0.3 samples. None of the samples show any frequency-dependent shift in their χ_AC_^′^ vs *T* curve to suggest a canonical spin glass.

The AC susceptibility data is shown in Figure 5. The χ_AC_^′^ vs *T* curves in panels (a) *x* = 0.1, (b) *x* = 0.2, and (c) *x* = 0.3 show no frequency-dependent shift. Neither were there any distinctive peaks in the imaginary component of the AC susceptibility (χ_AC_^″^ vs *T*) plotted in Supplementary Figure S16. As such, the expected AC signatures of a canonical spin glass are not observed in any of the samples.

### 3.4 Muon Spin Relaxation

**Figure 6:**
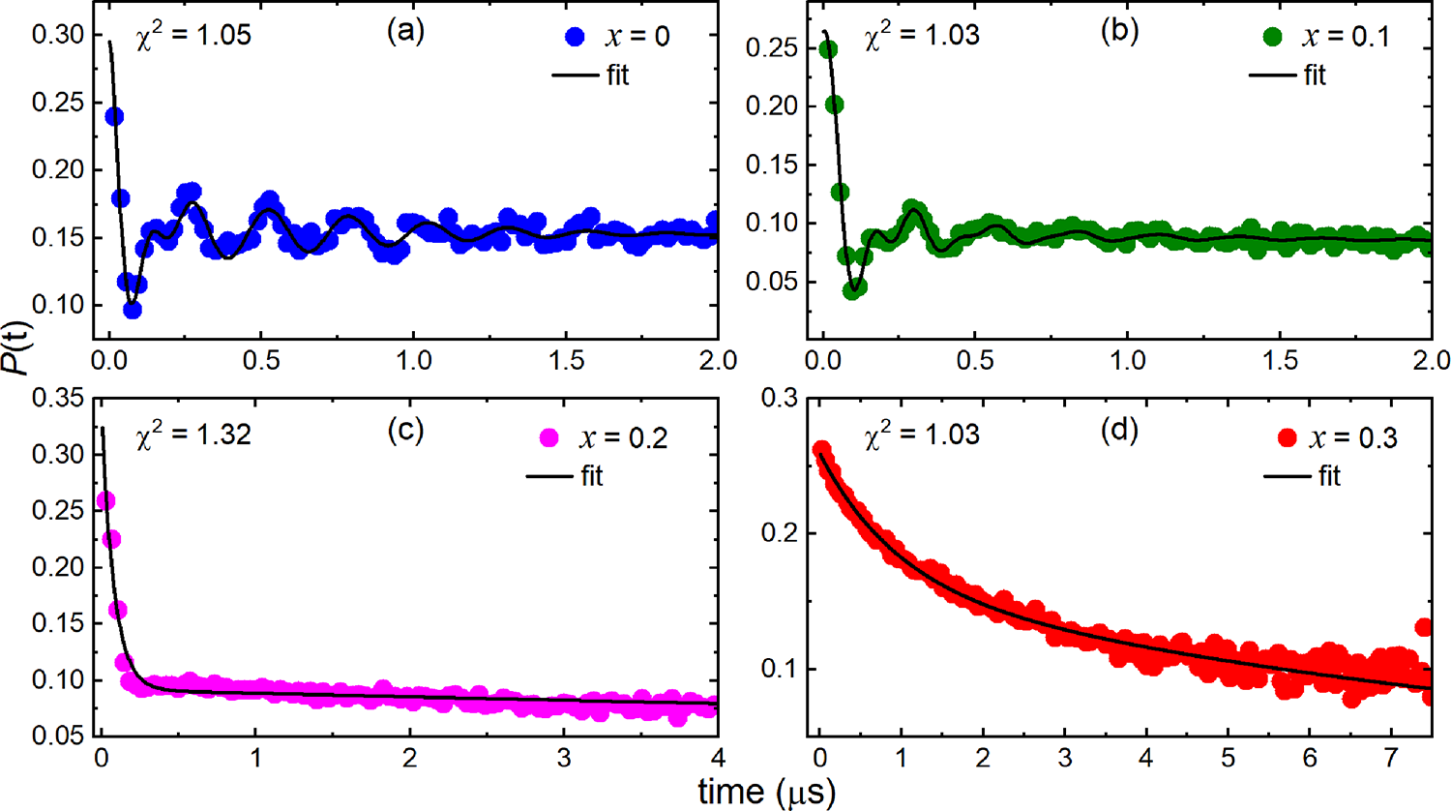
GPS ZF-μSR data of the (a) *x* = 0, (b) *x* = 0.1, (c) *x* = 0.2, and (d) *x* = 0.3 samples at 1.5 K. The black lines in the plots are the fits to the experimental data. Clear oscillations are observed for the *x* = 0 and *x* = 0.1 samples, demonstrating long-range order. Recovery of 1/3 of the initial asymmetry implies static ordering for *x* = 0.2, whereas exponential relaxation observed for *x* = 0.3 reflects a mostly dynamic magnetic environment. The goodness of fit (χ^2^) is shown in each panel.

Muon spin relaxation (μSR) experiments were performed on *x* = 0, 0.1, 0.2, and 0.3 to learn more about the local magnetic behavior. Previous measurements of Ba_2_CuTeO_6_ on ARGUS at the RIKEN-RAL using a pulsed muon source have identified a single oscillation of frequency *f* = 4.3 MHz in the ZF-μSR data at 2 K.^5^ Continuous muon sources such as PSI offer improved time resolution and can detect higher frequency oscillations compared to at pulsed sources. In this work, *x* = 0 was measured on GPS using a continuous PSI source, and the 1.5 K ZF-μSR data in Figure 6a shows that the signal is actually composed of two oscillations. The presence of two oscillations shows there are two muon stopping sites in Ba_2_CuTeO_6_. The two oscillations were described well using the polarization function in eq 1. This sums a Gaussian cosine and an exponential cosine (to describe the two oscillations) with an exponential background term.
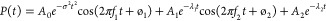
1

*A*_0_, *A*_1_, and *A*_2_ are the initial asymmetries, σ is the Gaussian decay rate, λ_1_ is the exponential cosine decay rate, and λ_2_ is the decay rate of the background term. *f*_1_ and *f*_2_ are the frequencies, and *ø*_1_ and *ø*_2_ are the phases of the respective oscillations. From the fit in Figure 6a, *f*_1_ = 3.81(1) MHz, *f*_2_ = 6.67(9) MHz, and the phases were zero in zero-field. The values of σ = 1.4(1) μs^–1^ and λ_1_ = 12.3(8) μs^–1^ show that muon relaxation is faster at the muon site described by the exponential cosine term.

Figure 6 compares the ZF-μSR data of *x* = 0 in panel (a) to the Ba_2_Cu_1 – *x*_Zn_*x*_TeO_6_ (b) *x* = 0.1, (c) *x* = 0.2, and (d) *x* = 0.3 data at 1.5 K. Clear oscillations are present in the *x* = 0.1 data in panel (b), showing that long-range magnetic order is still present. The oscillations were described poorly using the *x* = 0 polarization function in eq 1 (see Supplementary Figure S17). Instead, the *x* = 0.1 muon relaxation was better described using the polarization function involving Bessel functions in eq 2.
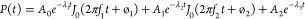
2

Here, two exponential zeroth-order Bessel functions *J*_0_(2π*f*_1_*t* + *ø*_1_) describe the two muon sites, and the exponential term describes the background. The fit in panel (b) of Figure 6 shows that eq 2 describes the muon relaxation of *x* = 0.1 well when the phases of the Bessel functions were non-zero (*ø*_1_= 33.9(4.2)° and *ø*_2_= −35.9(4.4)°). The use of Bessel functions implies an incommensurate magnetic structure where the non-zero phase arises from the infinite number of magnetically inequivalent muon sites.^37−39^ However, this behavior has also been observed in materials with significant disorder whose magnetic structures are commensurate.^8,40,41^ The *x* = 0 ZF-μSR data in Figure 6a was also fitted using eq 2 and is compared to the fit using eq 1 in Supplementary Figure S18. Both equations provided an adequate description of the muon polarization. The slight improvement in the fit using eq 2 implies that an incommensurate magnetic structure could also be plausible for *x* = 0. The ZF-μSR data for *x* = 0.1 at above 1.5 K in Figure S19 shows that the magnetic oscillations decay on warming and are no longer visible above 8 K. This indicates that 10% Zn^2+^ substitution lowered the transition temperature compared to *x* = 0 (*T*_N_= 14.1 K).

Panel (c) in Figure 6 shows the behavior of the *x* = 0.2 sample differs to *x* = 0.1. There are no oscillations to suggest long-range order. The muon polarization drops sharply at low times but quickly retains 1/3 of the initial asymmetry. Retention of 1/3 of the initial asymmetry suggests a static component to the muon relaxation as well as a dynamic component that leads to the sharp drop in the initial asymmetry. The combination of static and dynamic behavior can be phenomenologically described using the sum of a Gaussian dynamic Kubo–Toyabe function and an exponential as in eq 3, where *p*_z_(*t*) is the static Kubo–Toyabe function (eq 4), *v* is the muon hopping rate, δ is the width of the local field distribution, and λ the exponential decay rate.
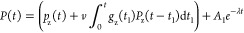
3
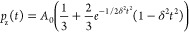
4

At 1.5 K, *v* is close to zero; therefore, the static Kubo–Toyabe function mainly contributes to *P*(*t*). This accounts for the 1/3 retention of the initial asymmetry. This shows that the spins are frozen at 1.5 K. ZF-μSR measurements at higher temperatures show that the muon hopping rate increases on warming (see Supplementary Figure S20), causing gradual loss of the 1/3 tail as the frozen static moments become dynamic. Note that the *x* = 0.2 muon relaxation also resembles a spin glass. However, fits using stretch exponentials did not derive a meaningful stretching exponent (i.e., β < 0.5) to support canonical spin glass behavior in agreement with the AC susceptibility data.

**Figure 7:**
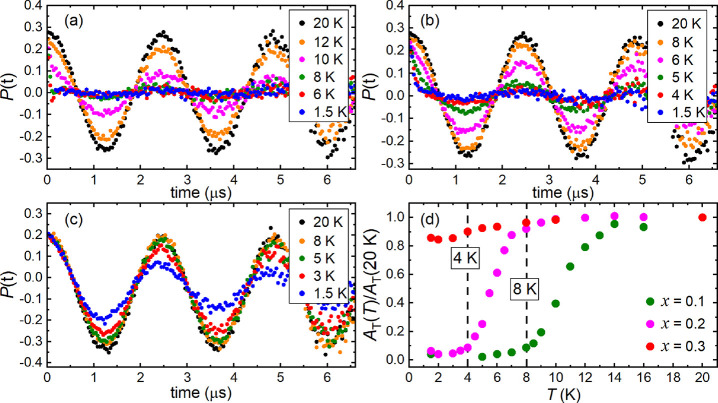
TF-μSR data for the (a) *x* = 0.1, (b) *x* = 0.2, and (c) *x* = 0.3 samples. TF-μSR data were collected at various temperatures between 1.5 and 20 K using a TF field of 30 G. Clear dampening is observed for the *x* = 0.1 and *x* = 0.2 samples, whereas the TF oscillations for *x* = 0.3 are only slightly damped at 1.5 K. Panel (d) plots the normalized TF asymmetry *A*_T_(*T*)/*A*_T_(20 K) for *x* = 0.1, 0.2, and 0.3 as a function of temperature, *T*. *A*_T_(*T*)/*A*_T_(20 K) was determined by fitting the TF oscillations using eq 6.

The 1.5 K ZF-μSR of *x* = 0.3 in panel (c) is exponential with no recovery of 1/3 of the asymmetry. The muon relaxation was described using two exponentials to reflect the two muon sites:
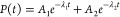
5ZF-μSR measurements on warming show that the high-temperature muon relaxation is quickly recovered as the dynamic fluctuations increase with temperature (see Supplementary Figure S21). Transverse field (TF)-μSR measurements were also performed on warming. Dampening of the TF-μSR oscillations indicates static magnetism as the muon spins begin to feel the effects of the internal fields and decouple from the TF field. While the majority of the Cu^2+^ moments in *x* = 0.3 are dynamic, slight dampening of the TF-μSR oscillations upon cooling in Figure 7c implies a small fraction of frozen spins. The TF-μSR asymmetry *A*_T_(*T*) was determined by fitting the TF oscillations using eq 6. The normalized *A*_T_(*T*)/*A*_T_(20 K) for *x* = 0.3 plotted in red in Figure 7d noticeably decreases below 10 K and suggests that ∼14% of the spins are frozen at 1.5 K.
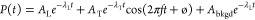
6

Figure 7 also shows the TF-μSR data of (a) *x* = 0.1 and (b) *x* = 0.2. Figure 7a shows complete dampening of the TF oscillations for *x* = 0.1. The *A*_T_(*T*)/*A*_T_(20 K) plot for *x* = 0.1 (green) in Figure 7d shows that long-range ordering is complete below 8 K, showing that Zn^2+^ lowered the ordering temperature of Ba_2_CuTeO_6_ (*T*_N_ = 14.1 K). The transition was gradual and occurred over a wider temperature range than might be expected for long-range ordering. The transition temperature was chosen as the point at which *A*_T_(*T*)/*A*_T_(20 K) plateaued to a constant value. Strong dampening was observed for *x* = 0.2 (Figure 7b). The value of *A*_T_(*T*)/*A_T_*(20 K) in Figure 7d plateaued to a constant value, indicating that freezing of the magnetic spins was complete below 4 K for *x* = 0.2 (shown in pink). It was noted that the TF oscillations were not completely damped at 1.5 K for *x* = 0.2 in Figure 7b, whereas they were in the *x* = 0.1 data in Figure 7a. This supports the existence of a small dynamic fraction (∼6%) at 1.5 K. It is also noted that the transitions in the *x* = 0.1 and *x* = 0.2 samples are also gradual. This is clearly shown in the plot in Figure 7d comparing the *A*_T_(*T*)/*A*_T_(20 K) data of *x* = 0.1, 0.2, and 0.3.

**Figure 8:**
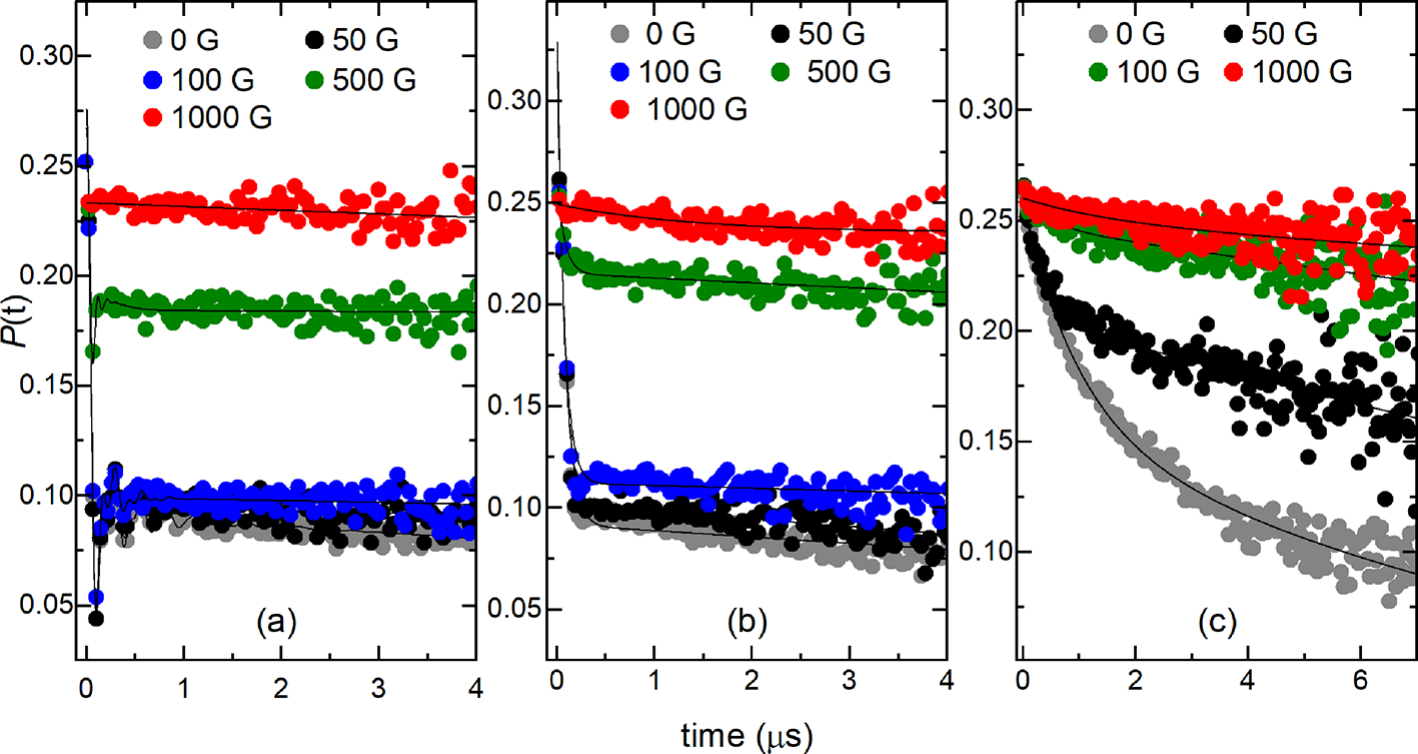
LF-μSR measurements of the (a) *x* = 0.1, (b) *x* = 0.2, and (c) *x* = 0.3 samples. LF measurements were performed at 1.5 K using LF fields of 50–1000 G.

Longitudinal field (LF)-μSR measurements of *x* = 0.1, 0.2, and 0.3 are compared in Figure 8. LF-μSR measurements indicate the field strength required to repolarize the muon spin in the direction of the LF field. Figure 8 shows the LF data of (a) *x* = 0.1 and (b) *x* = 0.2. For *x* = 0.1, suppression of the muon relaxation occurs above 100 G and complete repolarization occurs by 1000 G. The small suppression observed between 0 and 50 G represents decoupling from weak static nuclear spins. Larger LF fields are required to decouple electronic spins compared to nuclear spins. Repolarization requires weaker LF fields than might be expected for a long-range ordered sample owing to the weak Cu^2+^ moment and quantum fluctuations. The effects of the LF field can be seen at 100 G in the *x* = 0.2 dataset in Figure 8b. The stronger suppression between 50 and 100 G might represent decoupling from dynamic or static electronic spins as well as static nuclear spins. Similar to the *x* = 0.1 data, the largest changes occur above 100 G and the muon polarization is nearly completely recovered at 1000 G. The LF-μSR data for *x* = 0.3 in Figure 8c behaves differently. The muon polarization is gradually recovered as the LF field increases and is nearly complete at 1000 G. Suppression at 50 G likely represents decoupling from static nuclear spins, while the gradual recovery above 50 G resembles decoupling from dynamic electronic spins.

### 3.5 Heat Capacity

**Figure 9:**
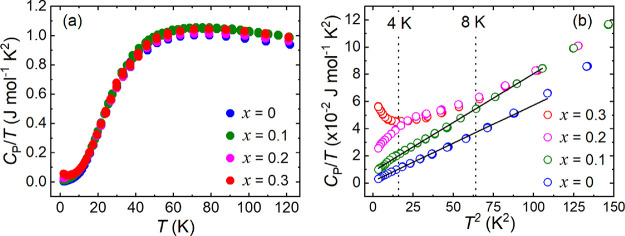
Heat capacity data for the *x* = 0, 0.1, 0.2, and 0.3 samples. Panel (a) shows the *C*_P_/*T* vs *T* data between 1.8 and 120 K. Panel (b) shows the low-temperature *C*_P_/*T* vs *T*^2^ data. Debye–Einstein fits for the *x* = 0 and *x* = 0.1 data between 1.8 and 109 K are shown by the black lines. The horizontal dotted lines at 8 and 4 K indicate the *T*_N_ of *x* = 0.1 and spin-freezing temperature of *x* = 0.2, respectively, determined from the TF-μSR measurements.

The zero-field heat capacity (*C*_P_/*T* vs *T*) data of the *x* = 0, 0.1, 0.2, and 0.3 samples is plotted in Figure 9a. As in previous reports, no clear Nèel transition can be observed for Ba_2_CuTeO_6_ at ∼14 K.^10,18^ The close proximity to the QCP creates quantum fluctuations that smear out the lambda (λ) ordering peak. The *C*_P_/*T* vs *T* curve of *x* = 0.1 is similar, with no evidence of a λ-peak about the *T*_N_ = 8 K indicated by the dotted line in the *C*_P_/*T* vs *T*^2^ plot in Figure 9b. This indicates that strong quantum fluctuations persist upon Zn^2+^ substitution. The *C*_P_/*T* vs *T*^2^ data of *x* = 0 and *x* = 0.1 between 1.8 and 109 K was linear and could be fitted well using the Debye–Einstein equation to determine the electronic (γ) and phonon ( β_D_) contribution to the heat capacity.
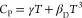
7

The γ-contribution was almost zero for *x* = 0 (γ = 1.4(1) mJ mol^–1^ K^–2^), in agreement with previous reports, where γ = 3.5(4) mJ mol^–1^ K^–2^.^18^ The value of γ was also close to zero for *x* = 0.1 (γ = 8.6(1) mJ mol^–1^ K^–2^). The low-temperature *C*_P_/*T* vs *T*^2^ data for *x* = 0.2 and *x* = 0.3 could not be fitted using eq 7. The *x* = 0.2 data shown in red in Figure 9b deviates slightly from linear behavior and has a slight bump at 4 K (dotted line). This is close to the spin-freezing transition identified in the TF-μSR measurements, so an association may be formed with this. The *x* = 0.3 data in black deviates from linear behavior as the temperature decreases, leading to an upturn below 4 K. The upturn indicates a clear change in the behavior between *x* = 0.2 and *x* = 0.3.

## 4 Discussion

Zn^2+^ was successfully substituted for Cu^2+^ within the spin ladder, forming a monoclinic Ba_2_Cu_1 – *x*_Zn_*x*_TeO_6_ solid solution. In-depth magnetic characterization using both bulk and local techniques showed that the spin ladder behavior changes as the Zn^2+^ concentration increases. Replacing magnetic Cu^2+^ with non-magnetic Zn^2+^ breaks local magnetic interactions in the system. Percolation theory can be used to explain the effects of such non-magnetic impurities on different spin systems.^20,21^ In this approach, below a critical impurity level known as the percolation threshold, the system contains one infinitely large cluster and smaller isolated clusters. Above this level, only isolated clusters remain. Therefore, the properties of these systems are different below and above the percolation threshold. For example, on a square, this threshold is 40.7%.^20^ The situation is different in one-dimensional systems such as spin chains and spin ladders. The percolation threshold is essentially zero as any finite level of impurities will break the system into isolated clusters. In a two-leg spin ladder, this occurs when two non-magnetic impurities neighbor each other, cutting the local ladder interactions.

The size of the clusters in spin ladders with non-magnetic impurities is still determined by percolation theory. We can understand the observed changes in the properties of Ba_2_CuTe_1 – *x*_Zn_*x*_O_6_ by considering how the cluster size changes with increasing *x*. The cluster size distribution intuitively depends on the impurity concentration *x* and is approximated as a geometric distribution:
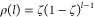
8ρ(*l*) is the probability of finding a cluster of *l* sites, which are mainly comprised of Cu^2+^*S* = 1/2 spins as well as potential isolated Zn^2+^ impurities that do not break the ladder interactions. ζ is the probability of breaking the ladder and is given by eq 9, where *x* is the impurity concentration that has a value between 0 and 1.
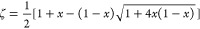
9

The average cluster size (*l̅*) is the expected value of the geometric distribution
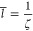
10

Any value of *x* leads to segmentation of the ladder into clusters; however, below a certain critical value (*x*_c_), the clusters are large enough to form long-range magnetic order. This agrees well with the result for *x* = 0.1. Using eqs 9 and 10, the average cluster size for *x* = 0.1 is calculated as *l̅* = 40 sites. There are clear oscillations in the μSR data below *T*_N_ = 8 K, showing that long-range order is retained. TF-μSR measurements in Figure 7 show that the transition is gradual. This can be explained by the distribution of cluster sizes, which are ordered at slightly different temperatures. The Weiss constant and position of *T*_max_ indicate slight weakening of the magnetic interactions. However, the susceptibility data could still be described using the isolated two-leg spin ladder model, where the reduced *J*_rung_/*J*_leg_ ratio also supports the weakening of the ladder interactions. There are remnants of the *T*_min_ feature from the “kink” in the low-temperature data. Also, like *x* = 0, the electronic contribution to the heat capacity was found to be near-zero, reflecting insulating behavior. There is some indication that the magnetic structure of *x* = 0.1 might be incommensurate. Low-temperature neutron diffraction would determine this, although it would require a high flux instrument given the very weak Cu^2+^ magnetic scattering. In any case, it is clear that the behavior of *x* = 0.1 closely resembles that of Ba_2_CuTeO_6_.

Curie–Weiss fitting shows further weakening of the interactions as the Zn^2+^ content increases. The *T*_max_ feature is suppressed, and the *T*_min_ upturn transitions into a large paramagnetic tail, suggesting the generation of “free” spins as Zn^2+^ breaks the Cu^2+^ interactions. Above *x* > 0.1, the two-leg spin ladder model began to deviate as the *T*_max_ was suppressed. This indicates that the Zn^2+^ concentration has exceeded the critical value beyond which the cluster size is too small to facilitate long-range magnetic order. For *x* = 0.2, the expected cluster size is only *l̅* = 11. There was no evidence of long-range order in the μSR data. Instead, the ZF-μSR measurements indicate frozen spins from the 1/3 recovery of the initial muon polarization below 4 K. This likely represents the formation of a long-range disordered static state, wherein the spins within clusters are statically ordered, but between clusters, the Cu^2+^ spins are long-range disordered. Similar to *x* = 0.1, the transition is gradual, reflecting the freezing of the different cluster sizes. However, the relaxation curve is not typical of static order, with a strong relaxing component at short times indicating that there is also a dynamic component to the muon relaxation. TF-μSR measurements suggest a small ∼6% fraction of dynamic electronic spins at 1.5 K. Decoupling of the ZF muon relaxation occurred at slightly weaker LF fields above 50 G compared to the >100 G required for *x* = 0.1, implying that dynamic electronic spins are present. The dynamic fraction arises from the portion of small clusters in the distribution in which there is too few spins to freeze.

The behavior further changes between *x* = 0.2 and *x* = 0.3. The heat capacity data of *x* = 0.3 shows an upturn that is not present in the *x* ≤ 0.2 data. There are a variety of plausible explanations for the low-temperature upturn, e.g., magnetic defects, spin fluctuations, or weak ferromagnetism.^42,43^ The *T*_max_ feature is suppressed in the χ vs *T* curve and can no longer be described by the two-leg spin ladder model. At 1.5 K, the ZF muon relaxation is mostly dynamic with only a small frozen fraction of spins (∼14%). The expected cluster size is *l̅* = 6 for *x* = 0.3; therefore, the small frozen fraction is likely to represent freezing of the small portion of large clusters in the distribution. Helium dilution fridge experiments would reveal whether this frozen fraction increases below 1.5 K. The LF-μSR data supports dynamic behavior, showing a gradual repolarization of the muon spins as the LF field increased. Therefore, as the average cluster size further decreases from *l̅* = 6 to *l̅* = 4 between *x* = 0.3 and *x* = 0.4, the system approaches a purely paramagnetic state. This leads to a Curie-like magnetic susceptibility for *x* ≥ 0.3, in which there is no *T*_max_ feature.

## 5 Conclusions

Ba_2_CuTeO_6_ has been shown to be a versatile structure, accommodating chemical substitution at both the magnetic Cu^2+^ site and non-magnetic *B*″ sites. Non-magnetic Zn^2+^ substitution at the Cu^2+^ site produced a Ba_2_Cu_1 – *x*_Zn_*x*_TeO_6_ solid solution (0 ≤ *x* ≤ 0.6). The results can be understood from the viewpoint of the percolation theory, whereby the Zn^2+^ impurities segmented the two-leg spin ladder into clusters. We observe three distinct types of behavior depending on the cluster size. For *x* = 0.1, the cluster size was large enough that long-range magnetic order was retained and the magnetic properties were similar to *x* = 0. As the cluster size is further reduced, the critical cluster size for long-range order is exceeded and a long-range disordered static state is proposed for *x* = 0.2. The behavior changes further between *x* = 0.2 and *x* = 0.3. Dynamic muon behavior was observed for *x* = 0.3, indicating a mostly paramagnetic state as the cluster size is too small to facilitate ordering or spin-freezing. This makes Ba_2_Cu_1-*x*_Zn_*x*_TeO_6_ an excellent model for studying non-magnetic impurities in two-leg spin ladders as the structural disorder (apart from that introduced by Zn^2+^) is low and the changes in magnetic behavior closely follow that expected for the percolation of a two-leg spin ladder.

## Supporting Information Available

Curie–Weiss fitting, imaginary AC susceptibility, analysis of the muon spin relaxation data, QMC isolated two-leg spin ladder model, and average cluster size for nonmagnetic dilution of a spin ladder (PDF)
CIF file for Ba_2_Cu_0.9_Zn_0.1_TeO_6_ (CIF)

## Supplementary Material

cm2c02939_si_001.pdf

cm2c02939_si_002.cif

## ABBREVIATIONS

QCP: quantum critical point
μSR: muon spin relaxation
QMC: Quantum Monte Carlo.
