# Supplementary material for: Partitioning the Two-Leg Spin Ladder in Ba2Cu1 – xZnxTeO6: From Magnetic Order through Spin-Freezing to Paramagnetism
Source: Chem Mater. 2023 Mar 22;35(7):2752–61. doi: 10.1021/acs.chemmater.2c02939 (PMC10100530; doi:10.1021/acs.chemmater.2c02939)
Supplement: Supplementary file 1 — cm2c02939_si_001.pdf [file cm2c02939_si_001.pdf]

Supporting information

"Partitioning the two-leg spin ladder in $Ba_2Cu_{1-x}Zn_xTeO_6$: from magnetic order through spin freezing to paramagnetism"

Charlotte Pughe[1], *Otto H. J. Mustonen*[1,2*], *Alexandra S. Gibbs*[3,4,5*], *Stephen Lee* [6], *Rhea Stewart* [6], *Ben Gade* [3], *Chennan Wang* [7], *Hubertus Luetkens*[7], *Anna Foster*[8], *Fiona C. Coomer*[9], *Hidenori Takagi*[5,10,11], *Edmund J. Cussen*[1*]

1. Department of Material Science and Engineering, University of Sheffield, Sheffield S1 3JD, United Kingdom
2. School of Chemistry, University of Birmingham, Edgbaston, Birmingham B15 2TT, United Kingdom
3. School of Chemistry, University of St Andrews, St Andrews, KY16 9ST
4. ISIS Pulsed Neutron and Muon Source, STFC Rutherford Appleton Laboratory, Didcot OX11 0QX, United Kingdom
5. Max Planck Institute for Solid State Research, Heisenbergstrasse 1, 70569 Stuttgart
6. School of Physics and Astronomy, University of St Andrews, St Andrews, KY16 9SS
7. Paul Scherrer Institute, Forschungsstrasse 111, 5232 Villigen PSI, Switzerland
8. Department of Chemistry, University of Sheffield, Sheffield S3 7HF, United Kingdom
9. Johnson Matthey Battery Materials, Reading RG4 9NH, United Kingdom
10. Department of Physics, University of Tokyo, Tokyo 113-0013, Japan
11. Institute for Functional Matter and Quantum Technologies, University of Stuttgart, 70569 Stuttgart, Germany

Corresponding Authors (*)

Edmund J. Cussen (e.j.cussen@sheffield.ac.uk)

Alexandra S. Gibbs (a.gibbs@st-andrews.ac.uk)

Otto H. J. Mustonen (ohj.mustonen@gmail.com)

Analysis of the HRPD neutron diffraction data

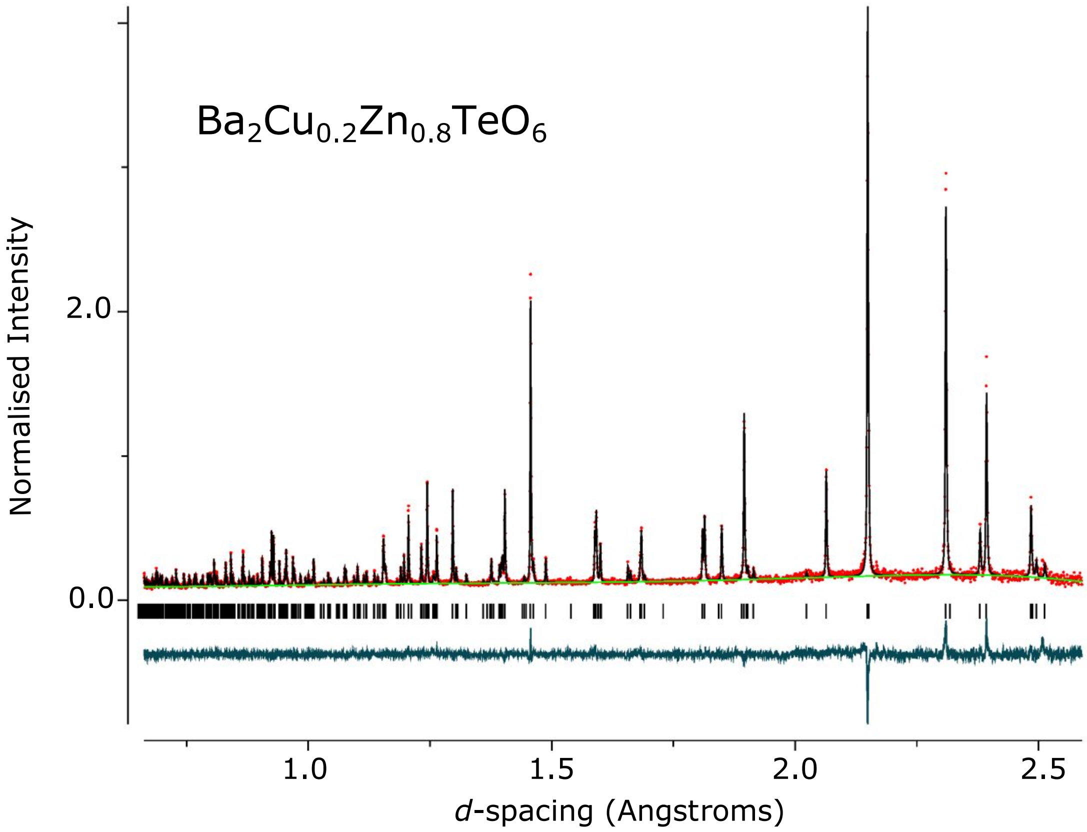

Fig. S1: The refined neutron diffraction pattern of $Ba_2Cu_{0.2}Zn_{0.8}TeO_6$ (x = 0.8). The red points indicate experimental data, the green line the fitted background, the black line the fit to the data and the dark blue line the difference profile. The black tick marks indicate reflection positions.

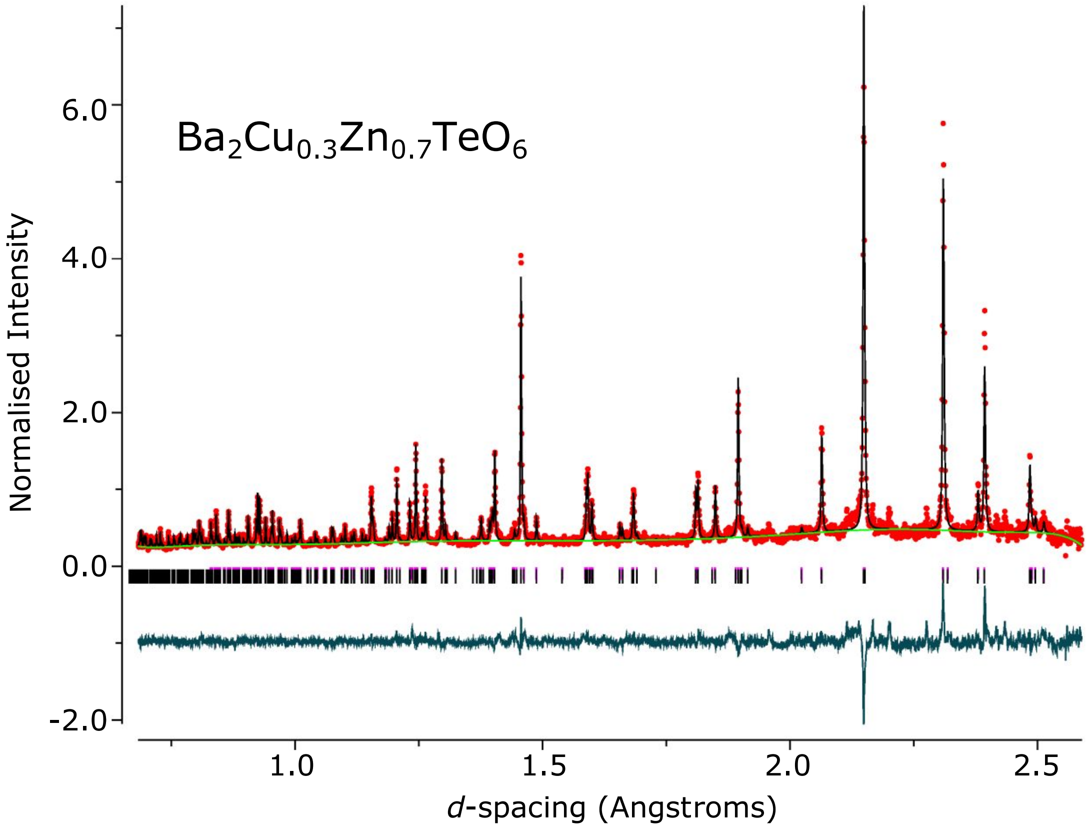

Fig. S2: The refined neutron diffraction pattern of $Ba_2Cu_{0.3}Zn_{0.7}TeO_6$ (x = 0.7). The red points indicate experimental data, the green line the fitted background, the black line the fit to the data and the dark blue line the difference profile. The black tick marks indicate reflection positions.

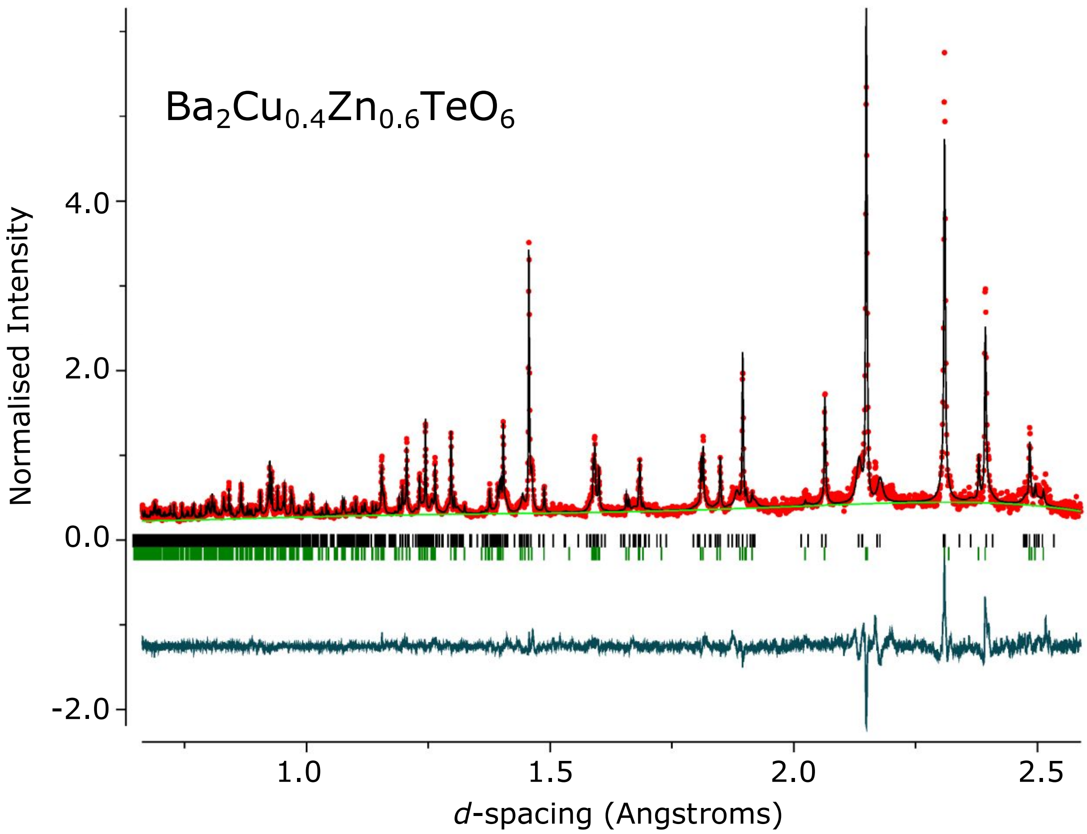

Fig. S3: The refined neutron diffraction pattern of $Ba_2Cu_{0.4}Zn_{0.6}TeO_6$ (x = 0.6). The red points indicate experimental data, the green line the fitted background, the black line the fit to the data and the dark blue line the difference profile. The black tick marks indicate reflection positions.

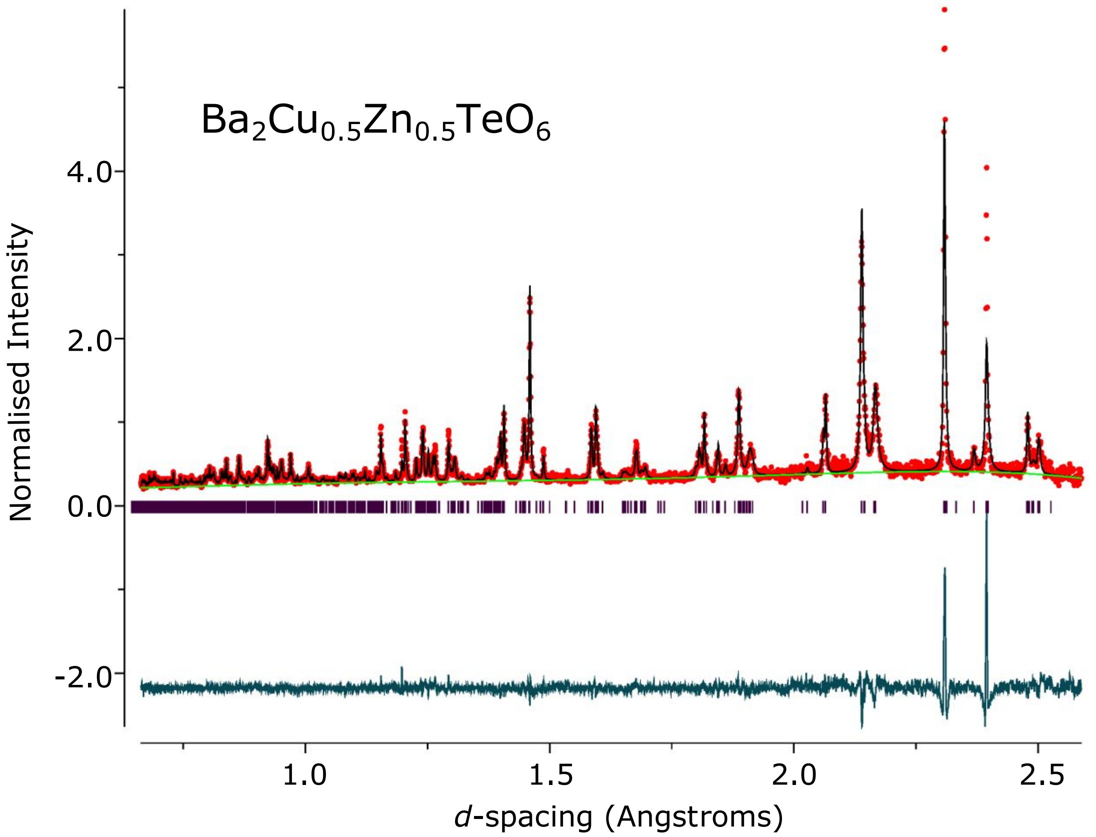

Fig. S4: The refined neutron diffraction pattern of $Ba_2Cu_{0.5}Zn_{0.5}TeO_6$ ($x = 0.5$). The red points indicate experimental data, the green line the fitted background, the black line the fit to the data and the dark blue line the difference profile. The black tick marks indicate reflection positions.

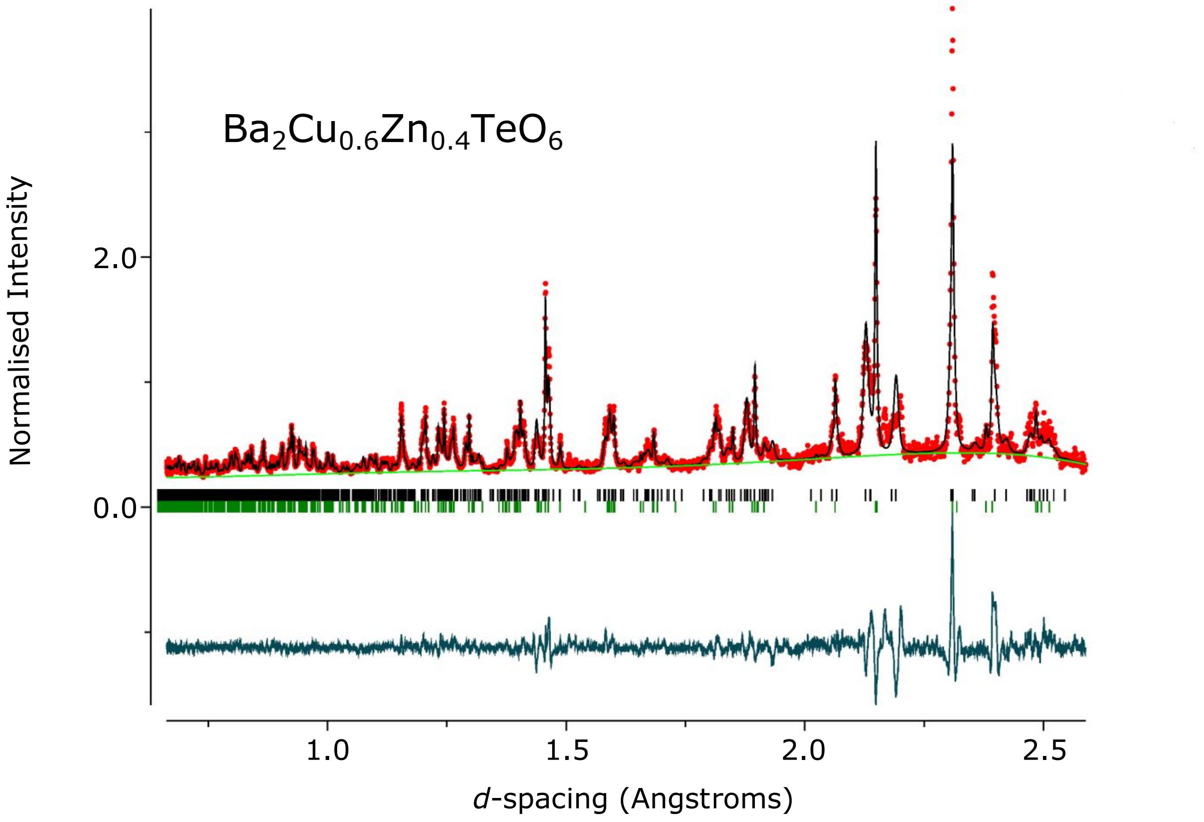

Fig. S5: The refined neutron diffraction pattern of $Ba_2Cu_{0.6}Zn_{0.4}TeO_6$ (x = 0.4). The red points indicate experimental data, the green line the fitted background, the black line the fit to the data and the dark blue line the difference profile. The black tick marks indicate reflection positions.

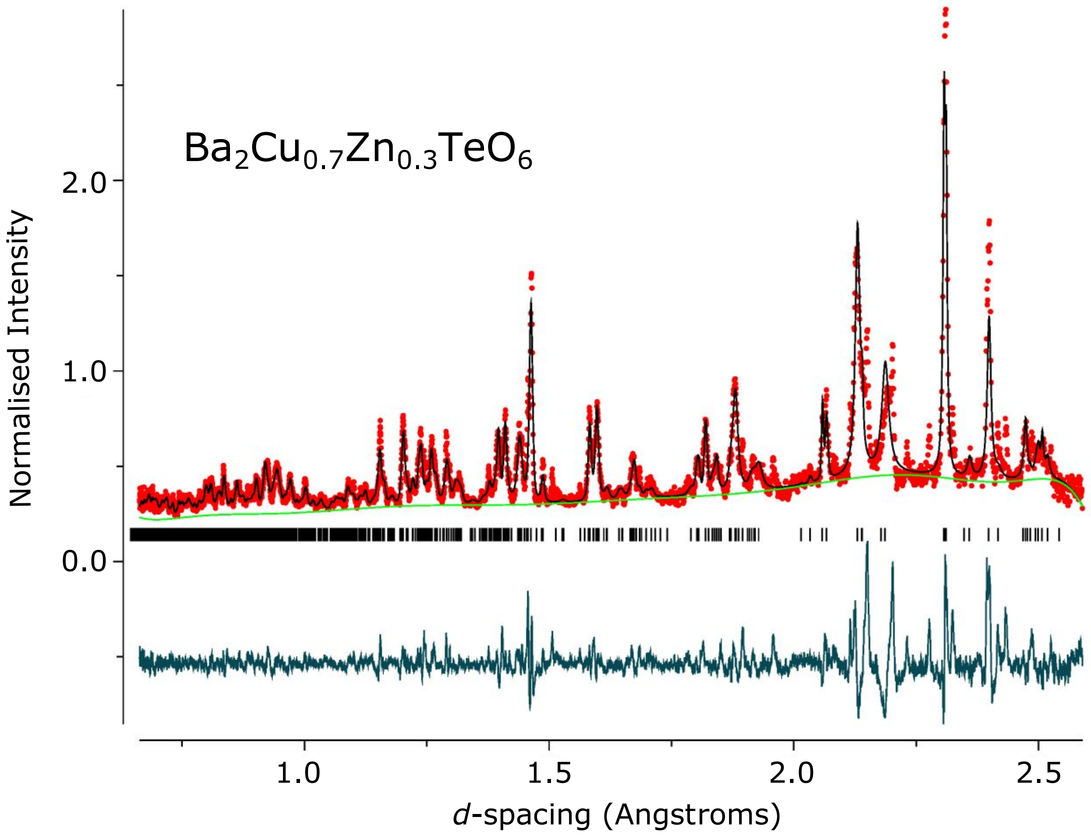

Fig. S6: The refined neutron diffraction pattern of $Ba_2Cu_{0.7}Zn_{0.3}TeO_6$ (x = 0.3). The red points indicate experimental data, the green line the fitted background, the black line the fit to the data and the dark blue line the difference profile. The black tick marks indicate reflection positions.

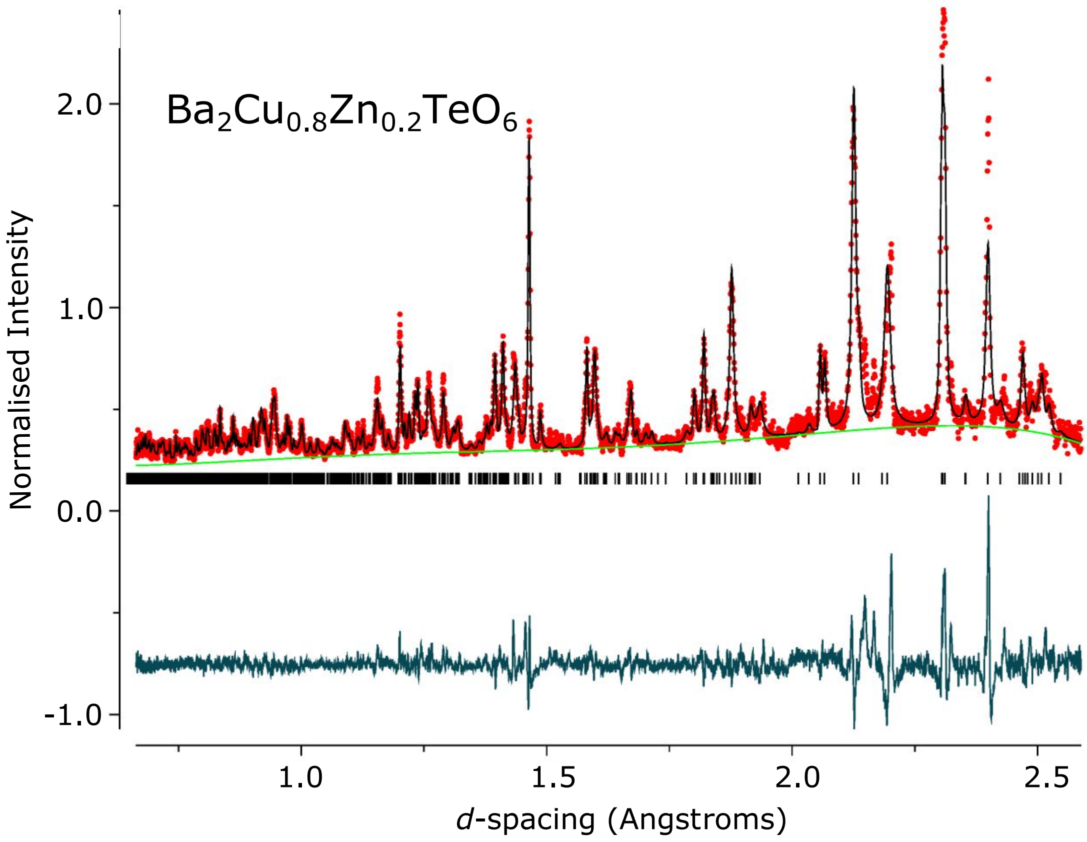

Fig. S7: The refined neutron diffraction pattern of $Ba_2Cu_{0.8}Zn_{0.2}TeO_6$ (x = 0.2). The red points indicate experimental data, the green line the fitted background, the black line the fit to the data and the dark blue line the difference profile. The black tick marks indicate reflection positions.

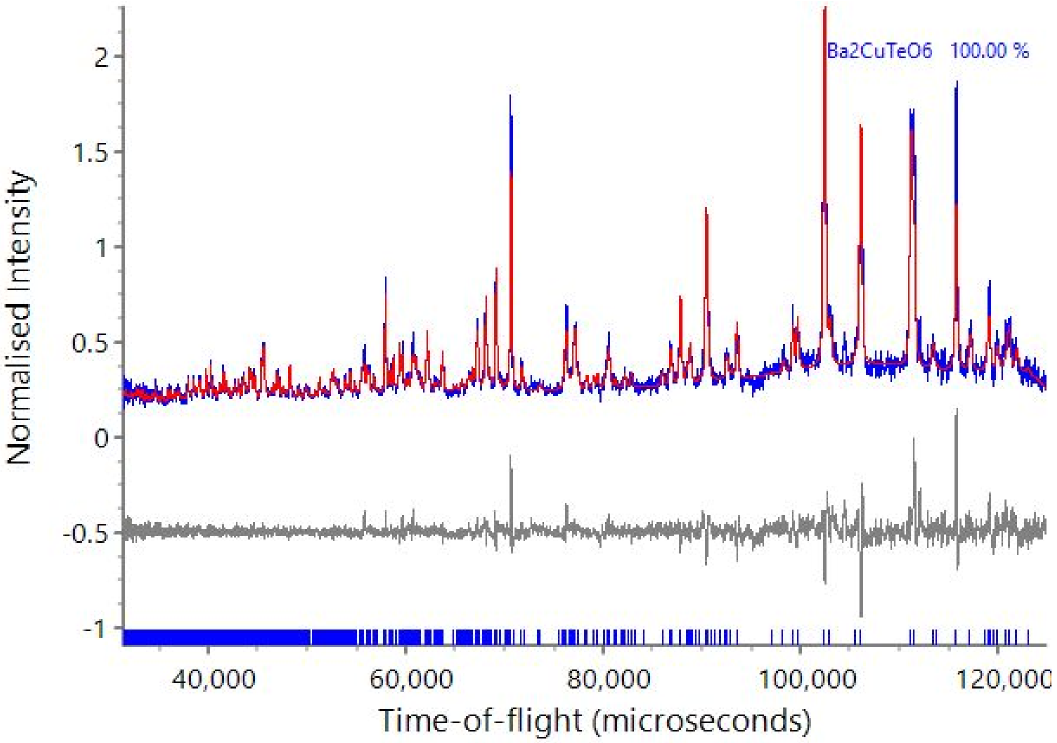

Fig. S8: The TOPAS-refined neutron diffraction pattern of $Ba_2Cu_{0.8}Zn_{0.2}TeO_6$ (x = 0.2). The blue lines indicate experimental data, the red line the fit to the data and the dark grey line the difference profile. The blue tick marks indicate reflection positions.

Table S1: Results of Rietveld refinement of HRPD powder neutron diffraction data from $Ba_2Cu_{0.1}Zn_{0.9}TeO_6$. Cu and Zn positions along with their U_{iso} values were constrained to be equal.

**$Ba_2Cu_{0.1}Zn_{0.9}TeO_6$**					
M_r = 563.45			c = 28.7079 (3) Å		
Trigonal, $R3m$			V = 843.60 (2) Å^3		
a = 5.82509 (5) Å			Z = 6		
Fractional atomic coordinates and isotropic or equivalent isotropic displacement parameters (Å^2)					
	x	y	z	U_{iso}	Occ. (<1)
Ba1	0.0	0.0	0.12785 (8)	0.0028 (6)	
Ba2	0.0	0.0	0.28068 (7)	0.0057 (6)	
Te1	0.0	0.0	0.0	0.0018 (7)	
Te2	0.0	0.0	0.5	0.0041 (6)	
Zn1	0.0	0.0	0.40298 (7)	0.0086 (6)	0.9
O1	0.15266 (12)	−0.15266 (12)	0.459880 (35)	0.0078 (4)	
O2	0.17794 (16)	−0.17794 (16)	0.62783 (4)	0.0139 (4	
Cu1	0.0	0.0	0.40298 (7)	0.0086 (6)	0.1

Table S2: Results of Rietveld refinement of HRPD powder neutron diffraction data from $Ba_2Cu_{0.2}Zn_{0.8}TeO_6$. Cu and Zn positions along with their U_{iso} values were constrained to be equal.

**$Ba_2Cu_{0.2}Zn_{0.80}TeO_6$**					
M_r = 563.27			c = 28.7119 (4) Å		
Trigonal, $R3m$			V = 843.62 (2) Å^3		
a = 5.82475 (6) Å			Z = 6		
Fractional atomic coordinates and isotropic or equivalent isotropic displacement parameters (Å^2)					
	x	y	z	U_{iso}	Occ. (<1)
Ba1	0.0	0.0	0.12766 (9)	0.0027 (7)	
Ba2	0.0	0.0	0.28070 (9)	0.0095 (7)	
Te1	0.0	0.0	0.0	0.0042 (8)	
Te2	0.0	0.0	0.5	0.0050 (8)	
Zn1	0.0	0.0	0.40326 (8)	0.0105 (7)	0.8
O1	0.15222 (15)	−0.15222 (15)	0.45990 (4)	0.0106 (5)	
O2	0.17793 (18)	−0.17793 (18)	0.62784 (4)	0.0152 (5)	
Cu1	0.0	0.0	0.40326 (8)	0.0105 (7)	0.2

Table S3: Results of Rietveld refinement of HRPD powder neutron diffraction data from $Ba_2Cu_{0.3}Zn_{0.7}TeO_6$. Cu and Zn positions along with their U_{iso} values were constrained to be equal (U_{iso} values were fixed to physically reasonable values for this site), U_{iso}s for sites containing barium were also constrained to be equal.

**$Ba_2Cu_{0.3}Zn_{0.7}TeO_6$**<br>$M_r$ = 563.08<br>Trigonal, *R*3*m*<br>*a* = 5.82416 (14) Å	*c* = 28.7102 (8) Å<br>*V* = 843.40 (5) Å^3<br>*Z* = 6

Fractional atomic coordinates and isotropic or equivalent isotropic displacement parameters (Å^2)

	x	*y*	*z*	U_{iso}	Occ. (<1)
Ba1	0.0	0.0	0.12671 (17)	0.0044 (9)	
Ba2	0.0	0.0	0.27977 (15)	0.0044 (9)	
Te1	0.0	0.0	0.0	0.0066 (15)	
Te2	0.0	0.0	0.5	0.0040 (14)	
Zn1	0.0	0.0	0.40354 (14)	0.0175 (15)	0.7
O1	0.1503 (3)	−0.1502 (3)	0.45987 (7)	0.0094 (9)	
O2	0.1755 (3)	−0.1754 (3)	0.62793 (8)	0.0143 (10)	
Cu1	0.0	0.0	0.40354 (14)	0.0175 (15)	0.3

Table S4: Results of Rietveld refinement of HRPD powder neutron diffraction data from $Ba_2Cu_{0.4}Zn_{0.6}TeO_6$. Cu and Zn positions along with their U_{iso} values were constrained to be equal (U_{iso}s were fixed to physically reasonable values for this site), U_{iso}s for sites containing the same element were also constrained to be equal.

**$Ba_2Cu_{0.4}Zn_{0.6}TeO_6$**<br>$M_r$ = 562.90<br>Monoclinic, *C*2/*m*<br>*a* = 10.1625 (10) Å<br>*b* = 5.7721 (6) Å	*c* = 10.1096 (11) Å<br>*β* = 108.608 (9)°<br>*V* = 562.02 (7) Å^3<br>*Z* = 4

Fractional atomic coordinates and isotropic or equivalent isotropic displacement parameters (Å^2)

	x	*y*	*z*	U_{iso}	Occ. (<1)
Ba1	0.1133	0.0	0.37293	0.0007 (17)	
Ba2	0.28222	0.0	0.85177	0.0007 (17)	
Te1	0.0	0.0	0.0	0.010 (2	
Te2	0.5	0.0	0.5	0.010 (2)	
Cu1	0.39684	0.0	0.21009	0.002	0.4
O1	0.13245	0.5	0.4053	0.0016 (7)	
O2	0.89185	0.72529	0.37059	0.0016 (7)	
O3	0.30723	0.5	0.88688	0.0016 (7)	
O4	0.04262	0.76369	0.87996	0.0016 (7)	
Zn1	0.39684	0.0	0.21009	0.002	0.6

Table S5: Results of Rietveld refinement of HRPD powder neutron diffraction data from $Ba_2Cu_{0.5}Zn_{0.5}TeO_6$. Cu and Zn positions along with their U_{iso} values were constrained to be equal, as were U_{iso} for Ba1 and Ba2.

$Ba_2Cu_{0.5}Zn_{0.5}TeO_6$

M_r = 562.72	c = 10.1208 (4) Å
Monoclinic, $C2/m$	β = 108.8722 (15)°
a = 10.1352 (4) Å	V = 561.98 (5) Å^3
b = 5.7899 (2) Å	Z = 4

Fractional atomic coordinates and isotropic or equivalent isotropic displacement parameters (Å^2)

	x	*y*	*z*	U_{iso}	Occ. (<1)
Ba1	0.1256 (7)	0.0	0.3805 (5)	0.0030 (9)	
Ba2	0.2833 (7)	0.0	0.8444 (5)	0.0030 (9)	
Te1	0.0	0.0	0.0	0.0076 (15)	
Te2	0.5	0.0	0.5	0.0029 (13)	
Cu1	0.4022 (4)	0.0	0.2110 (3)	0.0056 (12)	0.5
O1	0.1234 (7)	0.5	0.3907 (6)	0.0139 (18)	
O2	0.8876 (4)	0.7269 (7)	0.3753 (4)	0.0087 (10)	
O3	0.3100 (7)	0.5	0.8813 (7)	0.0174 (16)	
O4	0.0424 (5)	0.7633 (9)	0.8850 (4)	0.0137 (11)	
Zn1	0.4022 (4)	0.0	0.2110 (3)	0.0056 (12)	0.5

Table S6: Results of Rietveld refinement of HRPD powder neutron diffraction data from $Ba_2Cu_{0.6}Zn_{0.4}TeO_6$. Cu and Zn positions along with their U_{iso} values were constrained to be equal. Ba, Te ans (Cu/Zn) U_{iso}s were fixed at physically reasonable values.

$Ba_2Cu_{0.6}Zn_{0.4}TeO_6$

M_r = 562.53	c = 10.1101 (11) Å
Monoclinic, $C2/m$	β = 108.609 (9)°
a = 10.1629 (10) Å	V = 562.02 (7) Å^3
b = 5.7717 (6) Å	Z = 4

Fractional atomic coordinates and isotropic or equivalent isotropic displacement parameters (Å^2)

	x	*y*	*z*	U_{iso}	Occ. (<1)
Ba1	0.11637	0.0	0.37371	0.002	
Ba2	0.28147	0.0	0.85272	0.002	
Te1	0.0	0.0	0.0	0.002	
Te2	0.5	0.0	0.5	0.002	
Cu1	0.39765	0.0	0.20977	0.0022	0.6
O1	0.13161	0.5	0.40531	0.0031 (7)	
O2	0.89127	0.72697	0.37071	0.0031 (7)	
O3	0.3095	0.5	0.88304	0.0031 (7)	
O4	0.04287	0.75915	0.88137	0.0031 (7)	
Zn1	0.39765	0.0	0.20977	0.0022	0.4

Table S7: Results of Rietveld refinement of HRPD powder neutron diffraction data from $Ba_2Cu_{0.7}Zn_{0.3}TeO_6$. Cu and Zn positions along with their U_{iso} values were constrained to be equal. Ba and Te site U_{iso}s were constrained to be equal.

$Ba_2Cu_{0.70}Zn_{0.3}TeO_6$

M_r = 562.35
Monoclinic, *C*2/*m*
a = 10.1903 (13) Å
b = 5.7557 (8) Å
c = 10.1092 (13) Å
β = 108.395 (4)°
V = 562.6 (2) Å³
Z = 4

Fractional atomic coordinates and isotropic or equivalent isotropic displacement parameters (Å²)

	x	*y*	*z*	U_{iso}	Occ. (<1)
Ba1	0.1249 (9)	0.0	0.3808 (7)	0.0019 (15)	
Ba2	0.2715 (10)	0.0	0.8442 (8)	0.0019 (15)	
Te1	0.0	0.0	0.0	0.0019 (15)	
Te2	0.5	0.0	0.5	0.0019 (15)	
Cu1	0.4156 (7)	0.0	0.2108 (5)	0.010 (2)	0.7
O1	0.1205 (10)	0.5	0.3916 (9)	0.025 (3)	
O2	0.8910 (6)	0.7223 (10)	0.3746 (5)	0.0180 (19)	
O2	0.3090 (8)	0.5	0.8751 (8)	0.004 (2)	
O3	0.0483 (6)	0.7518 (13)	0.8912 (5)	0.0039 (15)	
Zn1	0.4156 (7)	0.0	0.2108 (5)	0.010 (2)	0.3

Table S8: Results of Rietveld refinement of HRPD powder neutron diffraction data from $Ba_2Cu_{0.8}Zn_{0.2}TeO_6$. Cu and Zn positions along with their U_{iso} values were constrained to be equal. Ba and Te U_{iso}s were constrained to be equal as were those for O.

$Ba_2Cu_{0.80}Zn_{0.2}TeO_6$

M_r = 562.17
Monoclinic, *C*2/*m*
a = 10.2121 (9) Å
b = 5.7402 (5) Å
c = 10.1017 (10) Å
β = 108.193 (3)°
V = 562.55 (14) Å³
Z = 4

Fractional atomic coordinates and isotropic or equivalent isotropic displacement parameters (Å²)

	x	*y*	*z*	U_{iso}	Occ. (<1)
Ba1	0.1298 (8)	0.0	0.3804 (6)	0.002	
Ba2	0.2762 (7)	0.0	0.8483 (6)	0.002	
Te1	0.0	0.0	0.0	0.002	
Te2	0.5	0.0	0.5	0.002	
Cu1	0.4071 (6)	0.0	0.2100 (5)	0.0143 (15)	0.8
O1	0.1308 (6)	0.5	0.3980 (6)	0.0071 (7)	
O2	0.8926 (4)	0.7250 (7)	0.3703 (4)	0.0071 (7)	
O3	0.3156 (6)	0.5	0.8686 (6)	0.0071 (7)	
O4	0.0524 (5)	0.7526 (10)	0.8943 (4)	0.0071 (7)	
Zn1	0.4071 (6)	0.0	0.2100 (5)	0.0143 (15)	0.2

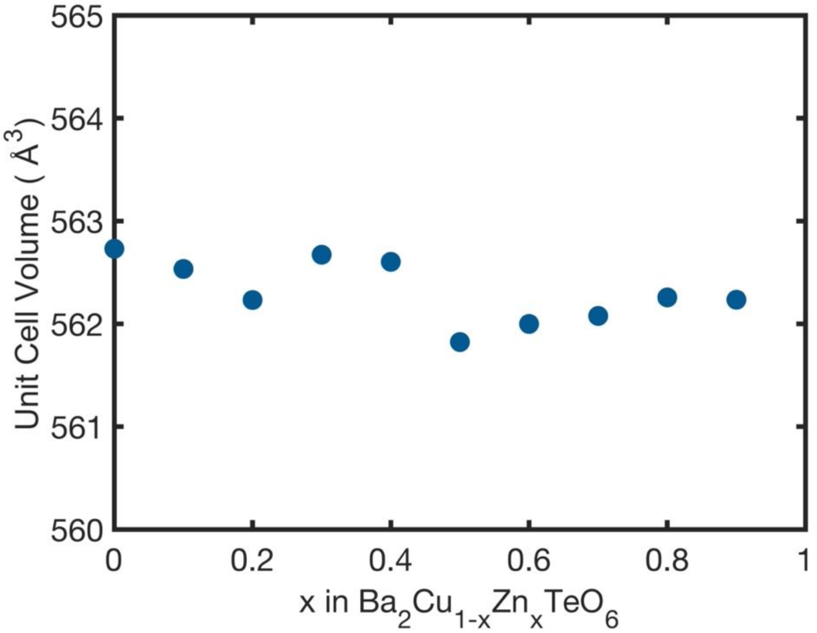

Fig. S9: The unit cell volume for $Ba_2Cu_{1-x}Zn_xTeO_6$ as a function of x based on TOPAS refinements. The unit cell volumes in the rhombohedral phase, for $x > 0.5$, are scaled by a factor of 2/3 in accordance with the change in x from 4 to 6 between the monoclinic and rhombohedral structures.

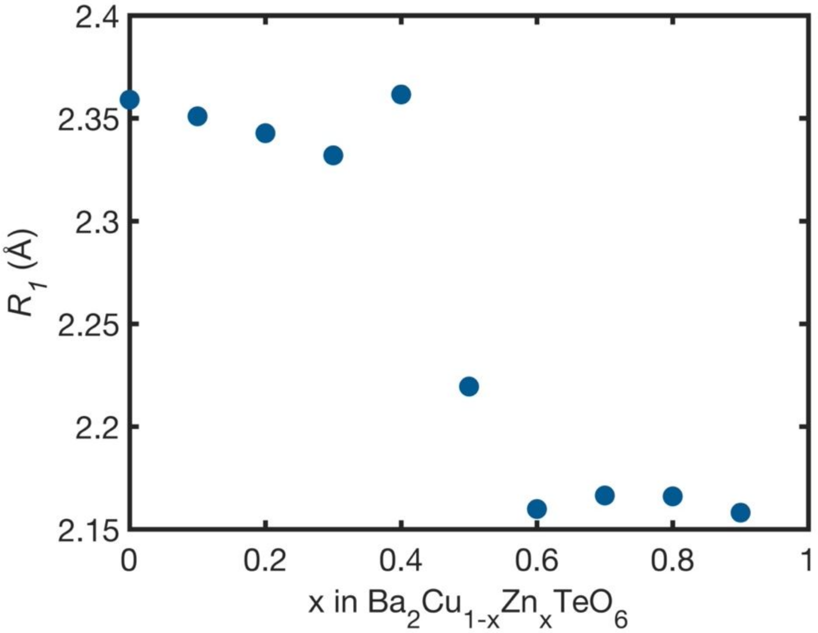

Fig. S10: The magnitude of the largest ellipsoidal principal axis for the CuO_6 octahedra, R_1, as a function of x based on TOPAS refinements. N.B. The value of R_1 and associated parameters for $x = 0.4$ is rather variable due to the difficulty in accurately fitting the data for this composition. This dataset has a more complex peak shape than the other compositions and the result is software dependent - GSAS appears to give a more smoothly decreasing dependence (when taking into account uncertainties in the PIEFACE-derived parameters, which are of the order of 0.05 Angstroms) whereas, for example TOPAS has an indication of a discontinuity around $x = 0.4$.[1–4] This is related to the details of the peak shape fit between the two sets of software. GSAS provided a better fit and therefore these values are quoted in the main manuscript. For completeness the TOPAS-derived parameters are plotted here in the Supplemental Material.

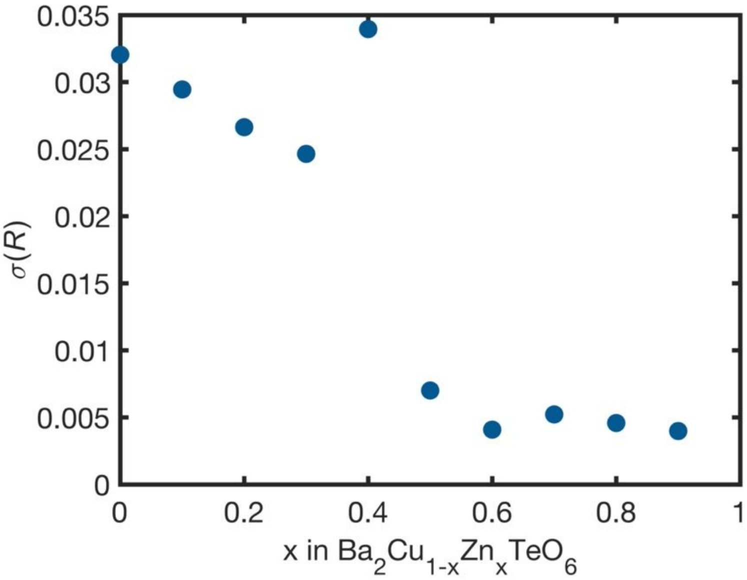

Fig. S11: The variance of the ellipsoidal principal axes as a function of *x* from TOPAS refinements.

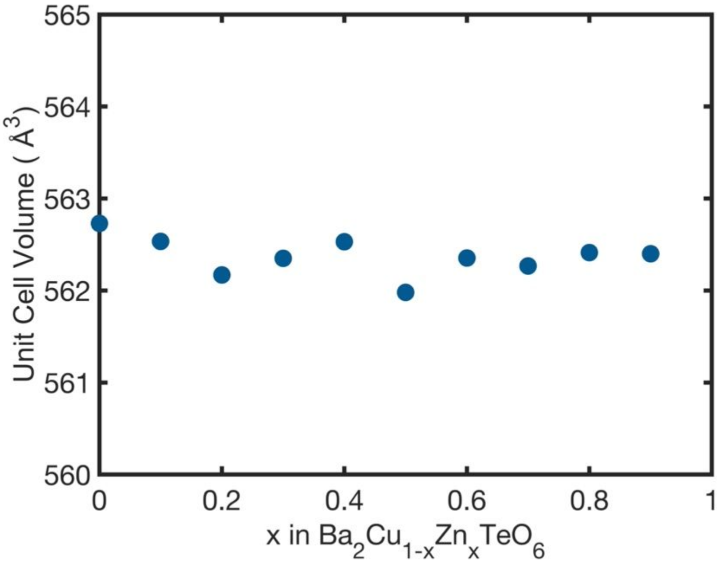

Fig. S12: The unit cell volume for $Ba_2Cu_{1-x}Zn_xTeO_6$ as a function of *x* based on GSAS refinements. The unit cell volumes in the rhombohedral phase, for $x > 0.5$, are scaled by a factor of 2/3 in accordance with the change in *x* from 4 to 6 between the monoclinic and rhombohedral structures.

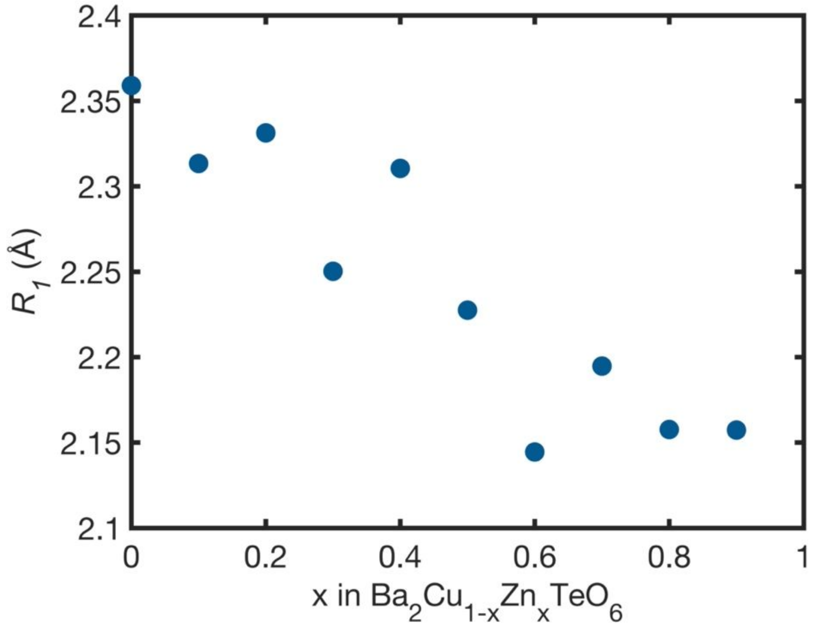

Fig. S13: The magnitude of the largest ellipsoidal principal axis for the CuO_6 octahedra, R_1, as a function of *x* based on GSAS refinements.

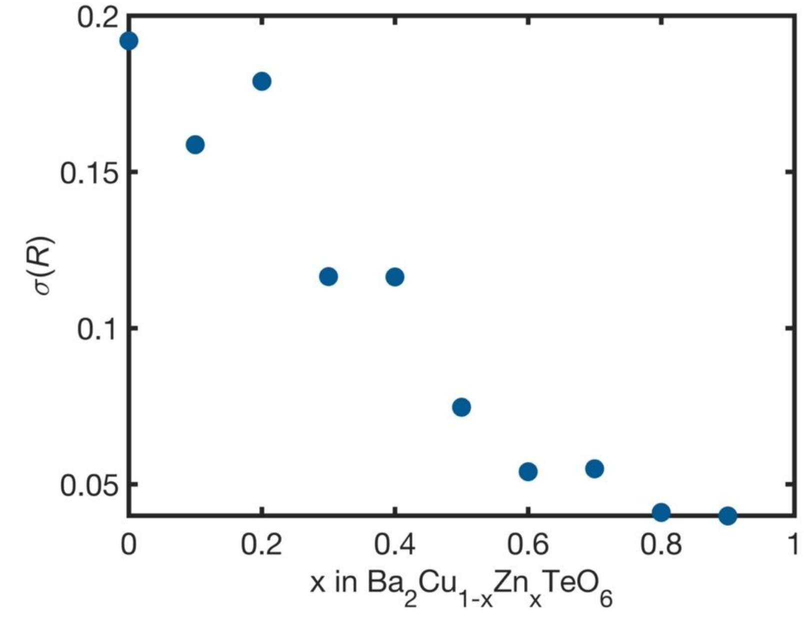

Fig. S14: The variance of the ellipsoidal principal axes as a function of *x* from GSAS refinements.

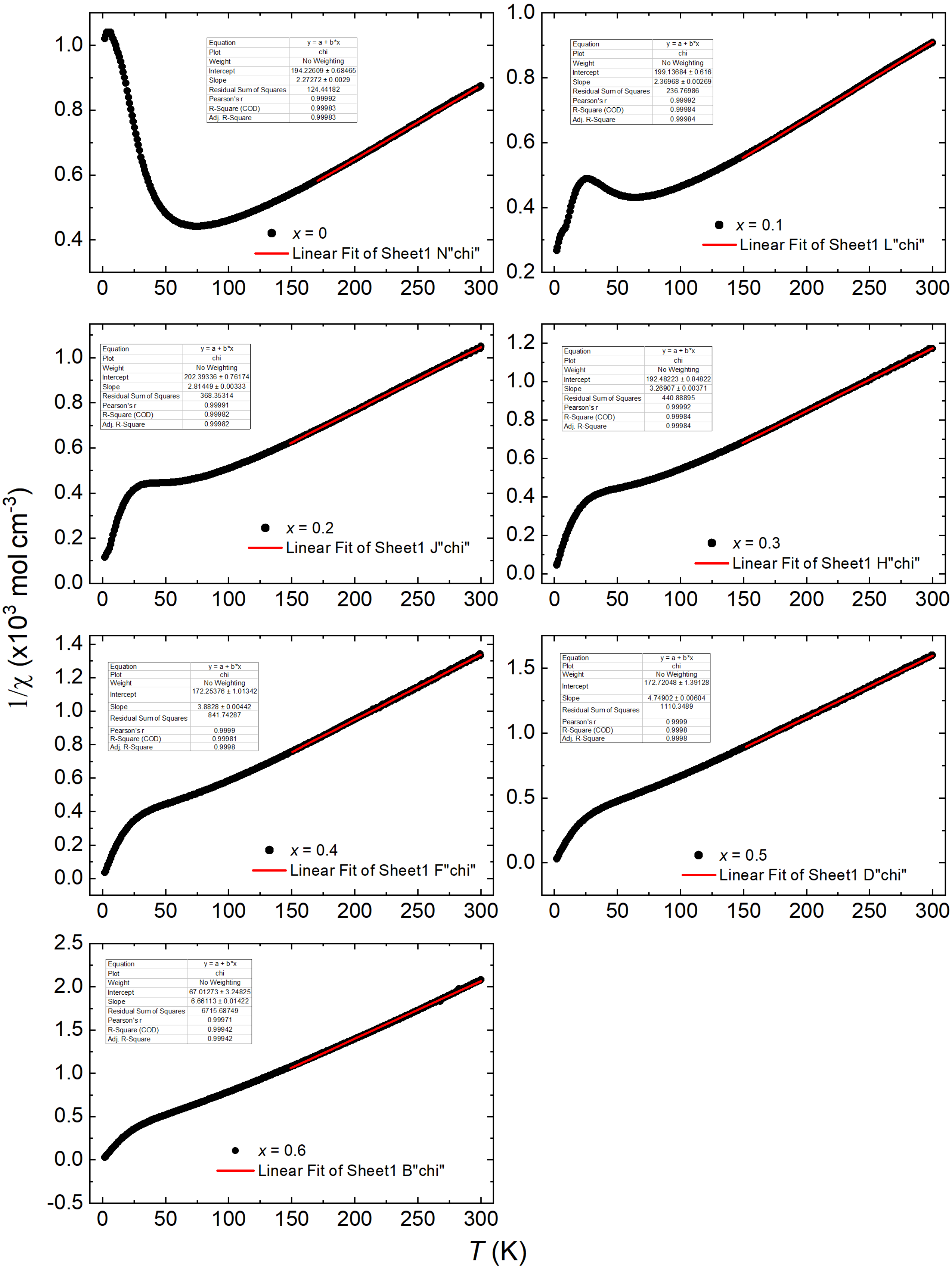

Fig. S15: Curie-Weiss fitting of the $Ba_2Cu_{1-x}Zn_xTeO_6$ ($0 \leq x \leq 0.6$) DC susceptibility data.

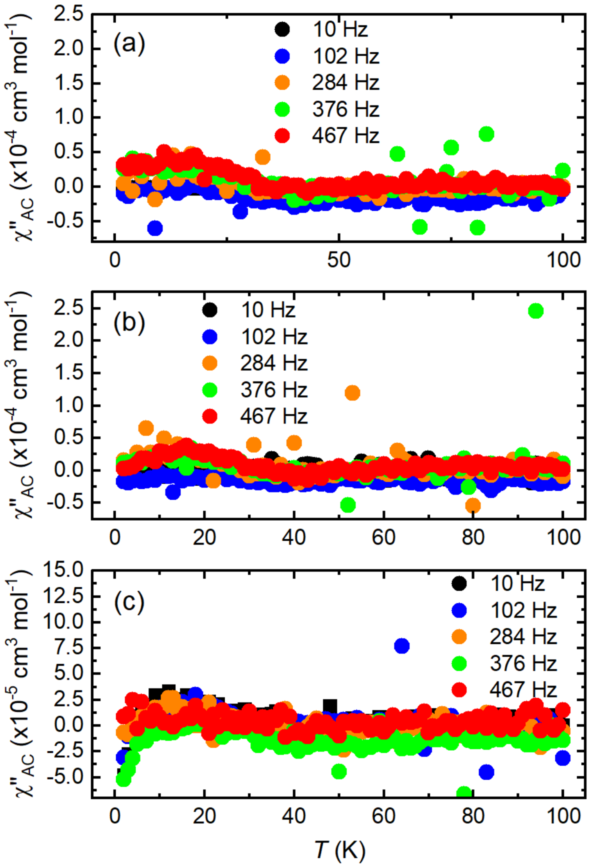

Fig. S16: The imaginary component (χ''_{AC}) of the AC susceptibility data in Fig. 5 plotted as a function of temperature for (a) x = 0.1, (b) x = 0.2 and (c) x = 0.3. There are no distinct peaks in the χ''_{AC} vs T data of any of the samples.

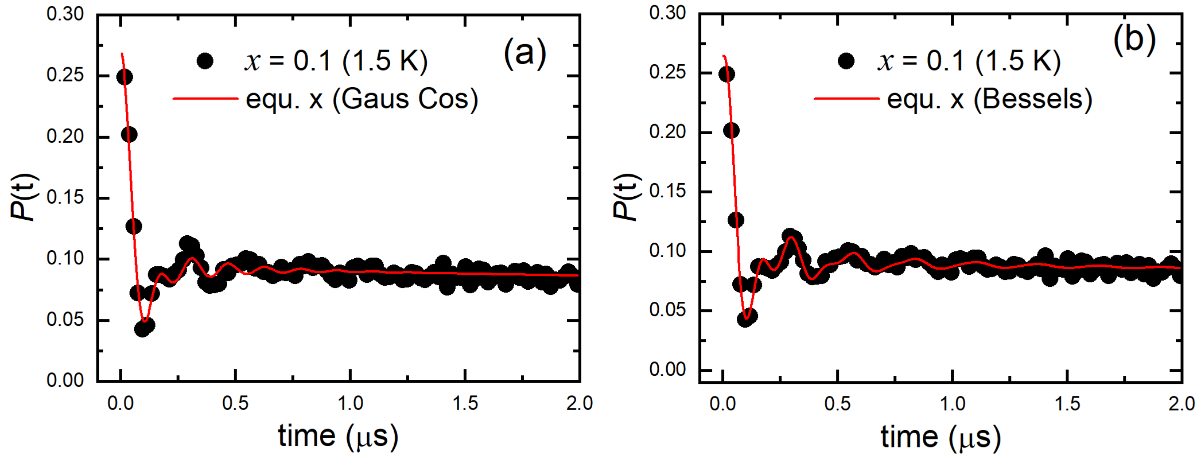

Fig. S17: Comparison of the fits to the 1.5 K x = 0.1 ZF-μSR data using equation 1 in panel (a) and equation 2 in panel (b). The fit using equation 1 involving Gaussian cosines provides a poor description of the ZF oscillations (χ^2= 1.111) compared to the fit using equation 2 involving Bessel functions (χ^2= 1.030).

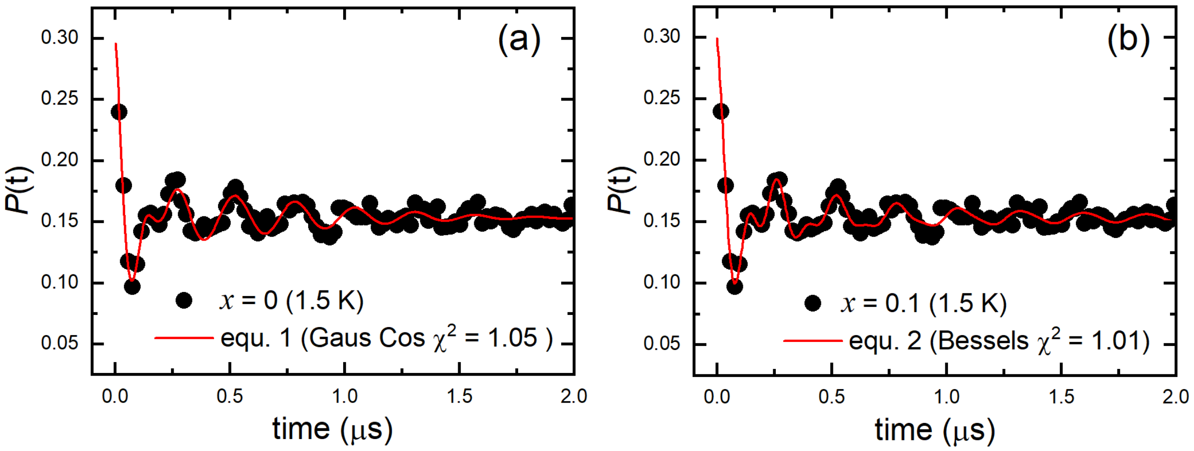

Fig. S18: Comparison of the fits to the 1.5 K *x* = 0 ZF-μSR data using equation 1 in panel (a) and equation 2 in panel (b). Comparing the fits shows equation 2 involving Bessel functions provides a slightly improved description of the muon relaxation. This might suggest the magnetic structure of *x* = 0 is also incommensurate.

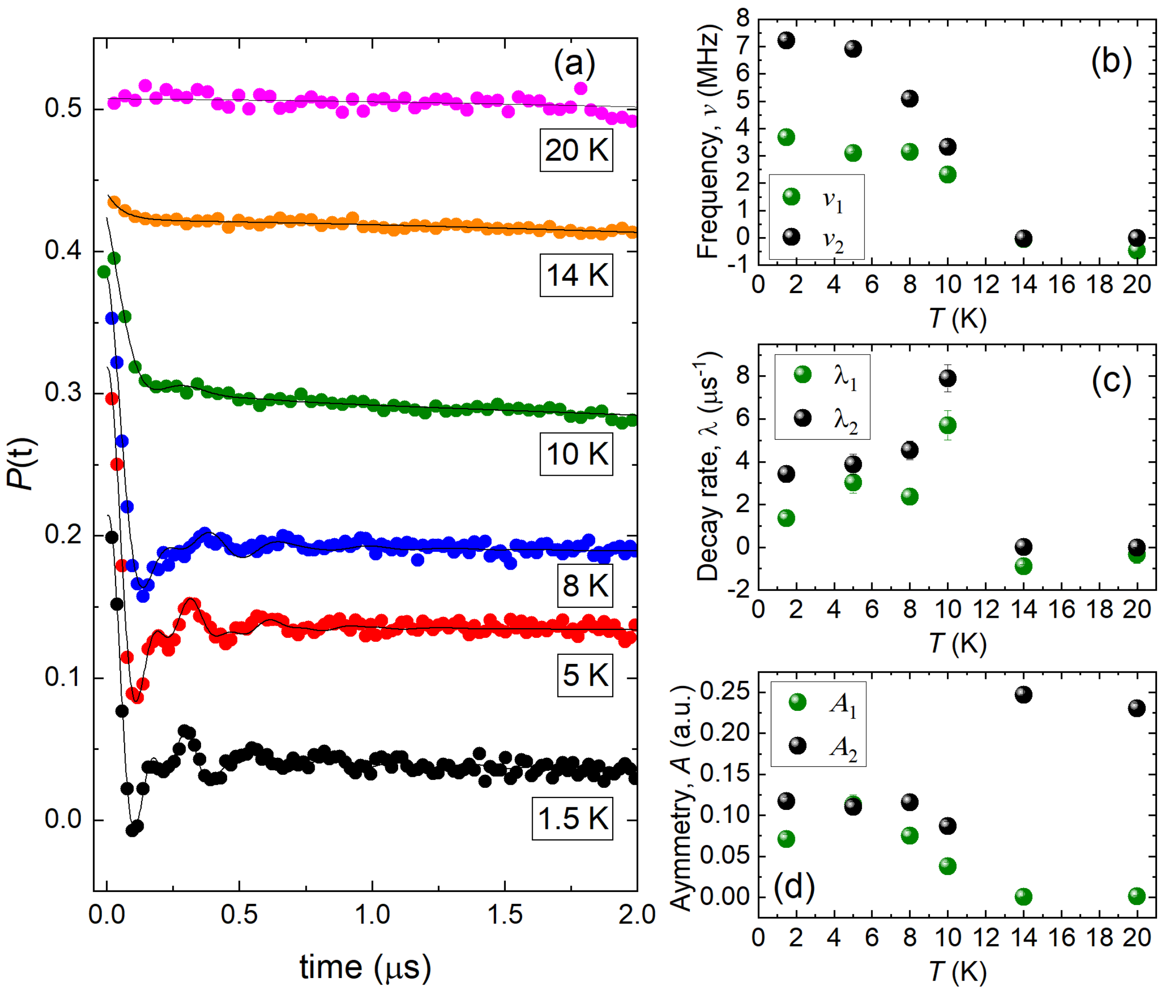

Fig. S19: The ZF-μSR data for x = 0.1 between 1.5-20 K. The low temperature magnetic oscillations decay with increasing temperature and are no longer clear above 8 K. Panels (b) to (d) show the values obtained for the fitting parameters as a function of temperature (T) using equation 2 including: (b) the values of the frequencies (ν_1 and ν_2); (c) the values of the decay rates (λ_1 and λ_2); and (d) the values of the asymmetries (A_1 and A_2).

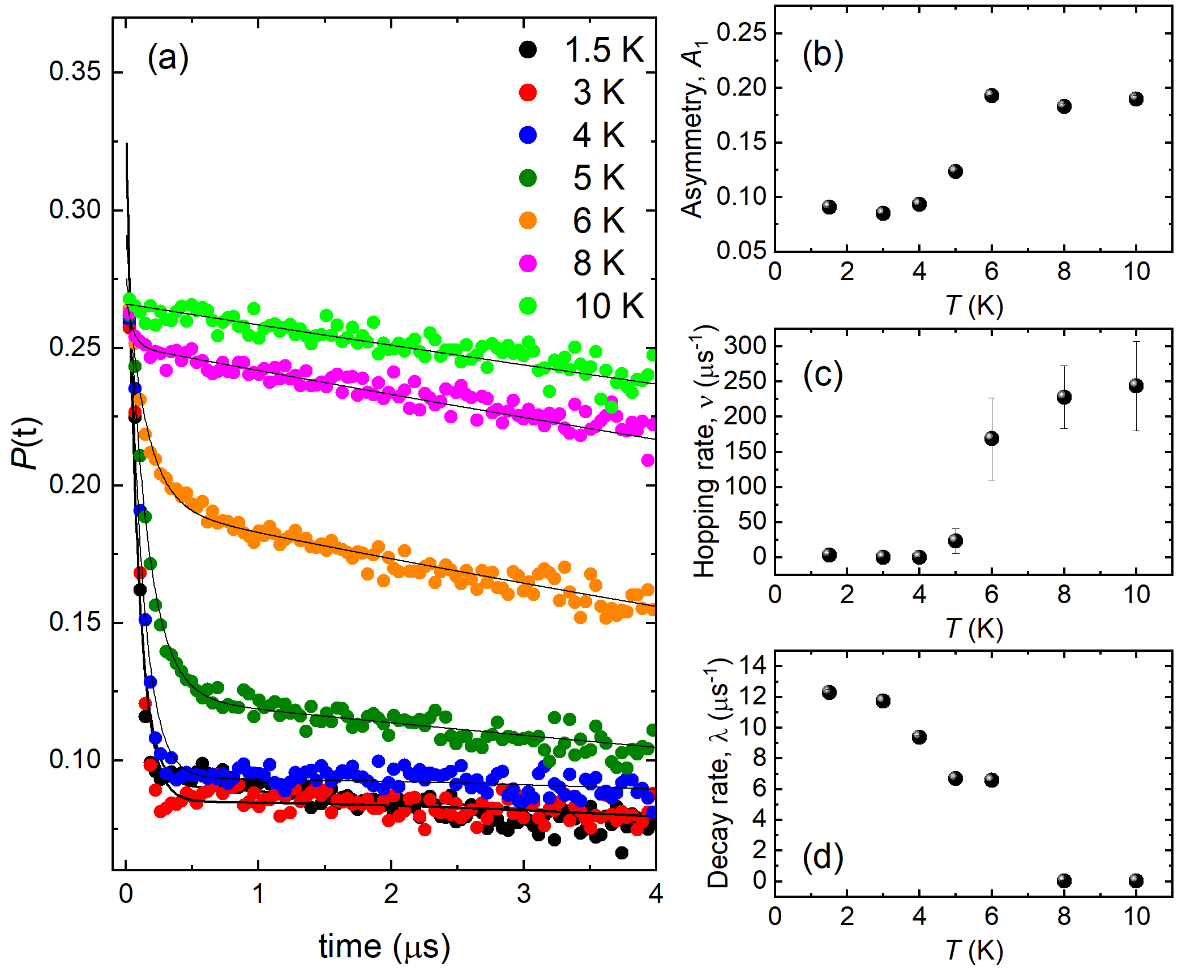

Fig. S20: The ZF-μSR data of x = 0.2 between 1.5-10 K. The data were fitted using the muon polarization function in equation 3. The parameters obtained from the fit are plotted as a function of temperature (T) in panels (b) - (d) and include: (b) the initial asymmetry (A_1), (c) the muon hopping rate (v) and (d) the exponential decay rate (λ).

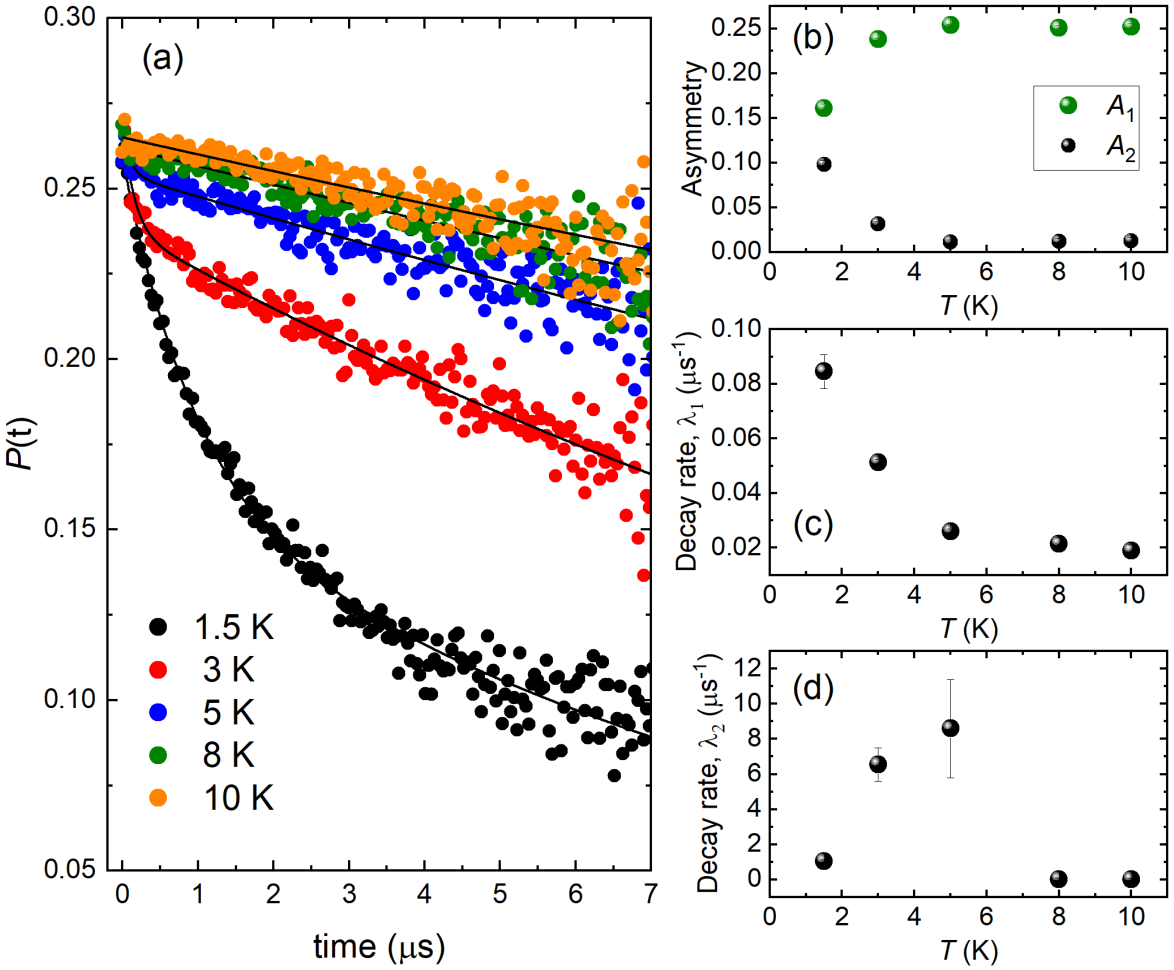

Fig. S21: The ZF-μSR data of x = 0.3 at temperatures between 1.5-10 K. The data were fitted using the muon polarization function in equation 5. The parameters obtained from the fit are plotted as a function of temperature (T) in panels (b), (c) and (d); where (b) shows the initial asymmetries (A_1 and A_2) and (c) and (d) are the decay rates, λ_1 and λ_2, of the respective exponential terms.

Isolated two-leg spin ladder model

The isolated two-leg spin ladder model from ref. is presented below. The model has three fitting parameters: J_{leg}, J_{rung}/J_{leg} and g and defines the molar magnetic susceptibility as:

$$\chi(T) = \frac{N_A g^2 \mu_B^2 e^{-\frac{\Delta J}{T}}}{4k_B T} P_6^6\left(\frac{T}{J_{leg}}\right) + \chi_0 \qquad \text{(1A)}$$

where,

$$\Delta = 0.4030\left(\frac{J_{rung}}{J_{leg}}\right) + 0.0989\left(\frac{J_{rung}}{J_{leg}}\right)^3 \qquad \text{(2A)}$$

and

$$P_6^6\left(\frac{T}{J}\right) = \frac{1 + \frac{N_1}{\left(\frac{T}{J_{leg}}\right)} + \frac{N_2}{\left(\frac{T}{J_{leg}}\right)^2} + \frac{N_3}{\left(\frac{T}{J_{leg}}\right)^3} + \frac{N_4}{\left(\frac{T}{J_{leg}}\right)^4} + \frac{N_5}{\left(\frac{T}{J_{leg}}\right)^5} + \frac{N_6}{\left(\frac{T}{J_{leg}}\right)^6}}{1 + \frac{D_1}{\left(\frac{T}{J_{leg}}\right)} + \frac{D_2}{\left(\frac{T}{J_{leg}}\right)^2} + \frac{D_3}{\left(\frac{T}{J_{leg}}\right)^3} + \frac{D_4}{\left(\frac{T}{J_{leg}}\right)^4} + \frac{D_5}{\left(\frac{T}{J_{leg}}\right)^5} + \frac{D_6}{\left(\frac{T}{J_{leg}}\right)^6}} \qquad \text{(3A)}$$

and

$$N_n = N_{n0} + N_{1n1}\left(\frac{J_{rung}}{J_{leg}}\right) + N_{1n2}\left(\frac{J_{rung}}{J_{leg}}\right)^2 + N_{1n3}\left(\frac{J_{rung}}{J_{leg}}\right)^3 \qquad \text{(4A)}$$

and

$$D_n = D_{n0} + D_{1n1}\left(\frac{J_{rung}}{J_{leg}}\right) + D_{1n2}\left(\frac{J_{rung}}{J_{leg}}\right)^2 + D_{1n3}\left(\frac{J_{rung}}{J_{leg}}\right)^3 + D_{1n4}\left(\frac{J_{rung}}{J_{leg}}\right)^4 + D_{1n5}\left(\frac{J_{rung}}{J_{leg}}\right)^5 + D_{1n6}\left(\frac{J_{rung}}{J_{leg}}\right)^6 + D_{1n7}\left(\frac{J_{rung}}{J_{leg}}\right)^7 + D_{1n8}\left(\frac{J_{rung}}{J_{leg}}\right)^8 + D_{1n9}\left(\frac{J_{rung}}{J_{leg}}\right)^9 \qquad \text{(5A)}$$

The values of the N_n and D_n coefficients were obtained from Table VII in ref [4] cited in ref. [5]. The powers of J_{rung}/J_{leg} up to 6 and 9 are only needed for D_2 with and D_3, respectively. In these equations, the ladder interactions (J_{rung} and J_{leg}) are expressed in units of Kelvin, K.

Average cluster size for nonmagnetic dilution of a spin ladder

Table S1 shows the average cluster size ($\bar{l}$) calculated using the equations (9) and (10) from ref. [6] for nonmagnetic dilution of a spin ladder, where ζ is the probability that the nonmagnetic impurity breaks the ladder and x is the nonmagnetic impurity concertation (i.e. amount of Zn^{2+}).

Table S9: Average cluster size obtained when partitioning a two-leg spin ladder using nonmagnetic impurity concentrations between 0 < x < 1.

x	ζ	$\bar{l}$ (avg cluster size, number of spins)
0.02	0.001	866.2
0.05	0.007	146.3
0.1	0.025	39.7
0.2	0.088	11.4
0.3	0.175	5.7
0.4	0.280	3.6
0.5	0.396	2.5
0.6	0.520	1.9
0.7	0.647	1.5
0.8	0.772	1.3
0.9	0.892	1.1
0.95	0.948	1.1
0.98	0.980	1.0

References

1. Larson, A. C. & Von Dreele, R. B. General Structure Analysis System (GSAS). *Los Alamos Natl. Lab. Rep. LAUR* 86–748 (2004).

2. Mccusker, L. B., Dreele, R. B. Von, Cox, D. E., Loue, D. & Scardi, P. Rietveld refinement guidelines. *J. Appl. Cryst.* **32**, 36–50 (1999).

3. Toby, B. H. EXPGUI, a graphical user interface for GSAS. *J. Appl. Crystallogr.* **34**, 210–213 (2001).

4. Coelho, A. A. TOPAS and TOPAS-Academic: An optimization program integrating computer algebra and crystallographic objects written in C++. *J. Appl. Crystallogr.* **51**, 210–218 (2018).

5. Johnston, D. C. *et al.* Magnetic Susceptibilities of Spin-1/2 Antiferromagnetic Heisenberg Ladders and Applications to Ladder Oxide Compounds. *arXiv:cond-mat/0001147 [cond-mat]* (2000).

6. Lavarélo, A., Roux, G. & Laflorencie, N. Magnetic responses of randomly depleted spin ladders. *Phys. Rev. B* **88**, 134420 (2013).
